# Surface engineering strategies for selectivity tuning and enhancement in photoelectrochemical biomass and CO_2_ valorization

**DOI:** 10.1039/d5sc02388b

**Published:** 2025-08-13

**Authors:** Yudhistira Tirtayasri Amrillah, Kaijian Zhu, Fani Rahayu Hidayah Rayanisaputri, Fita Widiyatun, Vivi Fauzia, Munawar Khalil, Fatwa F. Abdi, Ferry Anggoro Ardy Nugroho

**Affiliations:** a Department of Physics, Faculty of Mathematics and Natural Sciences, Universitas Indonesia Depok 16424 Indonesia f.a.a.nugroho@sci.ui.ac.id; b School of Energy and Environment, City University of Hong Kong 83 Tat Chee Avenue Kowloon Hong Kong S.A.R. China ffabdi@cityu.edu.hk; c Department of Chemistry, Faculty of Mathematics and Natural Sciences, Universitas Indonesia Depok 16424 Indonesia; d Institute for Advanced Sustainable Materials Research and Technology (INA-SMART), Faculty of Mathematics and Natural Sciences, Universitas Indonesia Depok 16424 Indonesia; e Advanced Materials and Molecular Synthesis Laboratory, Department of Chemistry, Faculty of Mathematics and Natural Sciences, Universitas Indonesia Depok 16424 Indonesia

## Abstract

Biomass and CO_2_ valorization constitutes a sustainable solution to mitigate global waste accumulation by converting biomass and CO_2_ into valuable chemicals and fuels. Among various conversion strategies, photoelectrochemical (PEC) systems have emerged as a promising approach due to their ability to drive redox reactions under mild conditions using solar energy. However, challenges such as poor selectivity, charge recombination, and inefficient light harvesting hinder the widespread adoption of PEC biomass and CO_2_ valorization. In efforts to push the concept into the practical realm, modifying the surface of the corresponding photoanodes has emerged as the most viable and effective approach. Acknowledging its importance, in this review, we thoroughly discuss various surface engineering strategies for enhancing and tuning PEC biomass and CO_2_ valorization selectivity. We open the discussion by introducing the fundamental principles of PEC processes, system configurations, and the critical role of surface properties in governing reaction pathways. Building on the previous discussions, common surface engineering strategies, particularly surface functionalization, crystal face tuning, defect engineering, and nanostructuring, are systematically reviewed for their ability to tailor surface properties and modulate the electronic structures of photoelectrodes. Crucially, we provide insights into the interplay between photoelectrode design and reaction dynamics responsible for the improvement and tunability of PEC biomass and CO_2_ valorization selectivity. By providing a comprehensive overview of recent advancements, this review aims to serve as a valuable resource for guiding future developments in PEC biomass and CO_2_ valorization.

## Abstract 2

This review extensively discusses various photoelectrode surface engineering strategies for tuning and enhancing photoelectrochemical biomass and CO_2_ valorization and provides an insightful perspective on the remaining challenges.

## Graphical abstract

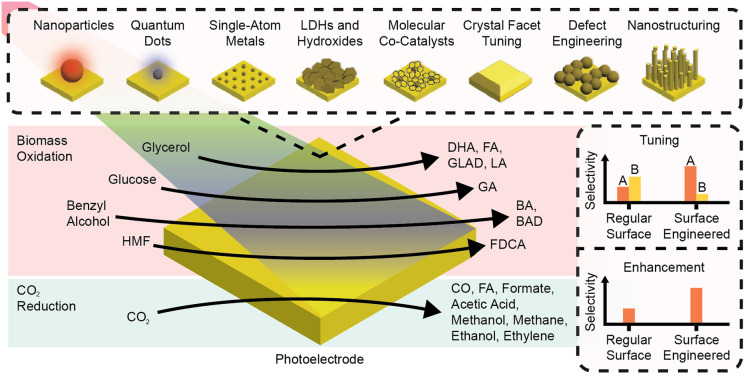

## 1. Introduction

The Industrial Revolution marked a turning point in human progress driving urbanization and industrialization as the primary pathways to economic and social development.^1^ However, rapid industrial and economic growth largely relies on finite fossil fuel resources, raising urgent concerns about energy security, environmental degradation, and sustainability.^2^ In response, global attention has turned toward renewable energy systems and circular economic models that reduce reliance on fossil fuels while mitigating waste accumulation and greenhouse gas emissions.^3,4^ In parallel with the decline of fossil fuel reserves, annual waste generation is projected to increase from 2.0 billion tonnes in 2018 to 3.4 billion tonnes by 2050.^5^ Without urgent intervention, the global annual cost of waste management could reach an alarming USD 640.3 billion.^6^ To address the environmental and economic burden of waste accumulation, it is imperative to develop recycling methods that not only reduce waste volumes but also convert biomass and CO_2_ into valuable chemicals and fuels, *i.e.*, upcycling.^7–9^ One promising approach is establishing a circular economic system centered on biomass and CO_2_ valorization to drive fuel production and waste reduction.^10^ In its broadest sense, biomass encompasses all organic matter produced *via* photosynthesis, whether directly harvested from plants or generated as by-products from industrial and municipal processes.^11^ In the context of waste reduction, biomass valorization plays a pivotal role by transforming organic waste materials, ranging from agricultural residues and food processing by-products to post-consumer waste, such as packaging, used vegetable oils, and demolition debris, into high-value products.^12^ Historically, agriculture has functioned as a circular economic system, repurposing livestock waste as manure, animal bedding, and soil-enriching by-products while utilizing crop residues to restore soil fertility.^13^ Expanding this circular model to incorporate diverse biomass and waste sources presents a viable strategy for mitigating both environmental challenges and energy shortages.

Scientific and technological advances have demonstrated that biomass can be efficiently transformed into valuable chemicals such as phenol and catechol, both of which have widespread use in various industries (*e.g.*, automotive, aerospace, building materials, electronics, consumer goods applications, fragrances and pharmaceuticals).^14–17^ However, conventional biomass valorization processes utilize a thermochemical approach that generally requires high temperatures, high pressures, and harsh reaction conditions, which severely limit reaction selectivity and overall process efficiency.^18,19^ In response to these limitations, photoelectrochemical (PEC) systems have emerged as a promising alternative.^20^ By harnessing solar energy to drive redox reactions under mild conditions, PEC technologies significantly reduce energy input and environmental impact while enabling the simultaneous production of value-added chemicals and fuels.^21,22^ Despite these advantages, PEC conversion faces significant challenges. In particular, rapid recombination of photogenerated charge carriers, inefficient light harvesting, and nonselective substrate activation can lead to the formation of mixed products rather than a single, desired target compound. For example, in PEC glycerol oxidation, which has attracted significant interest in recent years, glycerol waste, a byproduct from, *e.g.*, diesel production, can be converted into products such as dihydroxyacetone (DHA), formic acid (FA), glyceraldehyde (GLAD), glycolaldehyde (GCAD), and glycolic acid (GA), each with varying commercial values.^23–26^ Among these, DHA is particularly attractive due to its high market value and demand in the cosmetics, pharmaceutical, fine chemicals, and food industries.^27,28^ Yet, many high-efficiency methods tend to favor C–C bond cleavage, predominantly yielding lower-value compounds like FA rather than DHA. Similarly, PEC processes applied to 5-hydroxymethylfurfural (HMF) oxidation,^29^ glucose oxidation,^30^ methane conversion^31^ and CO_2_ reduction^32^ encounter challenges in achieving selective multi-carbon product formation—a necessity for generating high-energy-density fuels—due to difficulties in managing multi-electron transfer, suppressing charge recombination, and ensuring efficient charge transport.

**Fig. 1:**
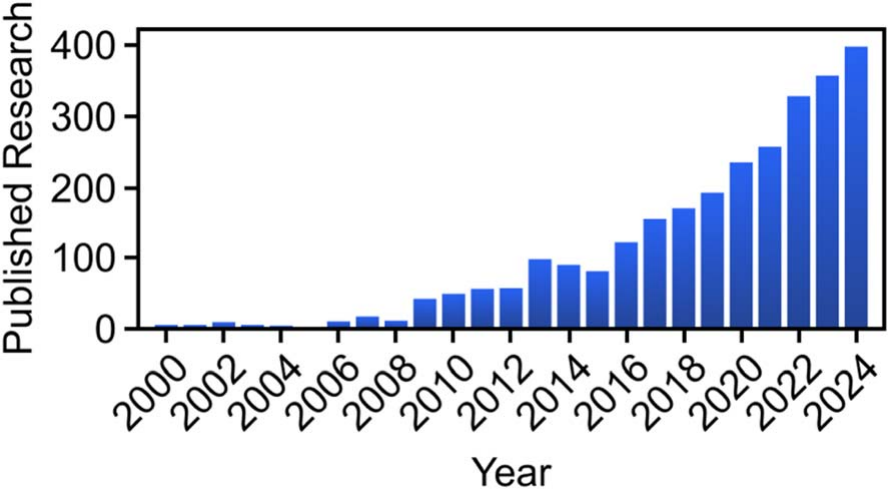
Number of Scopus-indexed research articles published from 2000 to 2024 on PEC biomass valorization. The research trend on PEC biomass valorization has been rising in recent years. The search string used in Scopus was “(photoelectrochemical OR photoelectrocatalytic OR PEC) AND (oxidation OR reduction OR conversion OR valorization) AND NOT (water OR sensor OR cell)”. Accessed March 22nd, 2025.

As the field of PEC biomass and CO_2_ valorization continues to grow rapidly (Fig. 1), surface engineering has emerged as a critical strategy to overcome challenges related to reaction selectivity, efficiency, and stability. This focus is understandable since surfaces are the literal sites where the reactions occur; it is thus logical to first modify them to generate certain chemical products selectively. Furthermore, surface engineering encompasses virtually unlimited strategies, for which minute details on the surface modification may give rise to contrasting effects. Specifically, by tailoring the catalyst surface at the nanometer and atomic scales, researchers have successfully modified the interfacial electronic structure, enhanced light absorption, and controlled the adsorption and activation of biomass-derived intermediates.^33,34^ Furthermore, surface functionalization approaches, such as incorporating nanoparticles,^35,36^ quantum dots,^37,38^ single-atom metals,^39^ layered double hydroxides and hydroxides,^40^ and molecular cocatalysts,^41–43^ not only enhance light harvesting efficiency and promote charge transport but also introduce more active reaction centers.^44,45^ In addition, crystal facet tuning^46^ and defect engineering^47,48^ enable precise adjustment of active sites to selectively promote specific reaction pathways.

While several reviews have addressed PEC biomass^23,49^ or CO_2_ (ref. 50–52) valorization individually, a comprehensive focus on surface engineering strategies that directly influence selectivity remains lacking. This review aims to fill this gap by systematically highlighting how specific surface engineering approaches, including surface functionalization, crystal facet tuning, defect engineering, and nanostructuring, can tailor surface properties. Furthermore, we aim to emphasize the interplay between photoelectrode surface design and reaction dynamics, providing mechanistic insights into how such modifications steer selective biomass and CO_2_ valorization. By integrating recent findings across both biomass and CO_2_ valorization systems and offering a cross-cutting perspective on selectivity control through surface engineering, this review offers a timely and unique contribution that can guide future advancements in the rational design of PEC systems for sustainable chemical production. Doing so, we begin by introducing the prospects of PEC biomass and CO_2_ valorization and its potential to significantly mitigate environmental and economic challenges. The principle of PEC valorization is then introduced along with typical PEC systems. Next, we discuss the importance of surface properties in PEC systems and their limitations, underscoring the necessity for surface engineering, followed by a detailed discussion on the principles, mechanisms, and the critical role of surface properties in PEC biomass valorization. Building upon this, state-of-the-art surface engineering strategies, including surface functionalization, crystal facet tuning, defect engineering, and nanostructuring approaches, that have been employed to improve and tune the selectivity of PEC biomass valorization are discussed. By highlighting how the interplay between catalyst design, defect engineering, and interfacial charge transfer is essential for overcoming kinetic and thermodynamic barriers, this review provides insights into future research directions that could drive the development of cutting-edge PEC technologies for sustainable biomass valorization.

## 2. PEC biomass valorization

Despite the rising interest in PEC biomass valorization,^20^ it still faces challenges such as low selectivity and limited efficiency, hindering its practical application and commercialization. Before discussing strategies targeted to alleviate these problems, it is therefore useful to first review the underlying principles of PEC biomass valorization, typical PEC systems, and the need for surface engineering for PEC biomass valorization. Understanding these factors will help one appreciate the various surface engineering strategies detailed in later sections.

### 2.1. Principles of PEC biomass valorization

PEC biomass valorization involves several key steps, beginning with light absorption using a photoelectrode. Initially, photons with energy higher than the bandgap of the semiconductor can be absorbed, exciting electrons from the valence band (VB, equivalent to the highest occupied molecular orbital, HOMO, in organic molecules) to the conduction band (CB, equivalent to the lowest occupied molecular orbital, LUMO, in organics), leaving behind holes in the VB. Here, the absorption coefficient plays a critical role in photon capture efficiency, as a higher absorption coefficient allows for thinner films and shorter charge transfer distances. Beyond the intrinsic properties of the material, strategies such as nanostructuring can introduce light-trapping effects, further enhancing light absorption efficiency.^53^ The next step involves photogenerated charge separation and recombination. In inorganic semiconductors, free electrons and holes are typically separated by internal electric fields, which can arise from various mechanisms such as band bending or facet engineering.^54^ However, due to their low dielectric constant, organic semiconductors often require additional layers^55^ or specific stacking configurations^56^ to facilitate exciton dissociation into free charge carriers. Charge separation typically occurs within hundreds of femtoseconds to a few picoseconds, while charge recombination occurs on a timescale ranging from picoseconds to microseconds.^57^ The lifetime of active charge carriers is influenced by various factors during transport, including particle–particle grain boundaries and defects. In general, materials with high conductivity, mobility, and charge carrier density enable more effective charge transport, reducing recombination losses. Finally, the transferred charge carriers accumulate on the electrode surface, driving chemical reactions. Since catalytic reactions occur over milliseconds to seconds, a significant portion of charge carriers may deactivate before contributing to the reaction. Additionally, the adsorption modes of different reactant molecules influence charge transfer from the electrode to the reactant, impacting biomass conversion kinetics and selectivity. As a result, the electrode/electrolyte interface plays a crucial role in determining reaction selectivity and efficiency.

**Fig. 2:**
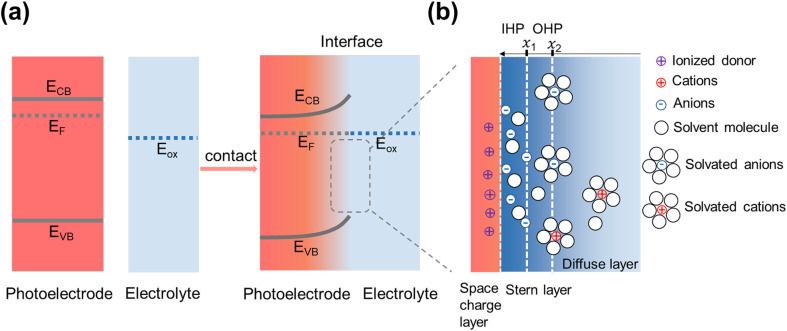
Mechanism of photoelectrode/electrolyte interface equilibration. (a) The band energetics of a semiconductor/liquid before and after contact. (b) Model of the double-layer structure of an n-type semiconductor electrode in contact with the electrolyte under equilibrium conditions.

When a semiconductor is immersed in an electrolyte, charge transfer occurs due to the potential difference between the semiconductor's Fermi level (*E*_F_) and the electrolyte's redox potential (*E*_redox_). Upon equilibration, *E*_F_ aligns with *E*_redox_, leading to band bending and the formation of a space charge layer (Fig. 2a). Taking an n-type semiconductor used for oxidation reactions as an example, the downward shift of *E*_F_ induces upward band bending, which results in hole accumulation within the space charge layer. On the electrolyte side, an electrical double layer, consisting of the Stern layer and the Gouy-Chapman diffuse layer, forms at the interface. The Stern layer, located closest to the semiconductor, contains adsorbed ions and solvated molecules and is further divided into the inner Helmholtz plane (IHP) and the outer Helmholtz plane (OHP). The IHP corresponds to the position of the electrical centers of adsorbed ions at a distance *x*_1_, while the OHP represents the position of solvated molecules or ions, which can only approach the semiconductor up to a distance *x*_2_ (see Fig. 2b).^58^ In this regard, changes in electrolyte composition, such as variations in cations and anions, can significantly influence the distribution of adsorbed ions in the IHP and solvated species in the OHP, thereby affecting both reaction activity and selectivity. Several studies have reported the effects of different cations and anions on, *e.g.*, glycerol oxidation.^59,60^ Beyond ion type, potential drops across the Stern layer and Gouy–Chapman diffuse layer must also be considered in biomass valorization. The concentration of biomass feedstock plays a crucial role in influencing ionic conductivity, electrolyte viscosity, and electrode surface coverage—all of which impact conversion efficiency and selectivity. For example, studies have shown that variations in glucose concentration distinctly affect mass transport and electron transfer kinetics.^61^ In the case of glycerol oxidation, the peak current was found to increase as glycerol concentration rose from 0.5 to 1.0 M but decreased at concentrations above 1.0 M, a decline attributed to insufficient OH^−^ coverage on the electrode surface.^62^ Another study investigated how varying glycerol concentrations influenced selectivity, finding that higher concentrations favored the production of C_3_ products.^63^

### 2.2. Structure of a typical photoelectrochemical system

**Fig. 3:**
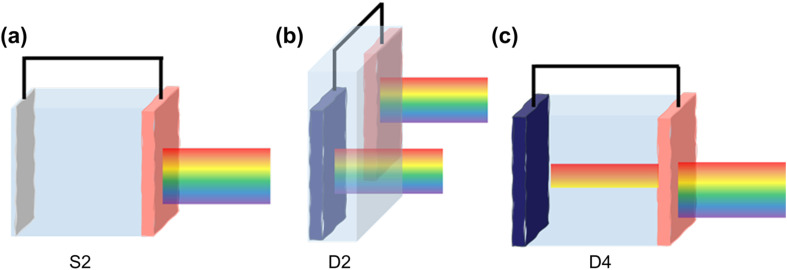
Configurations of a typical PEC system. (a) S2, the system employs a single bandgap PEC cell with only one electrode absorbing light. (b) D2, the system utilizes a dual bandgap PEC cell with two electrodes being illuminated side-by-side. (c) D4, a dual bandgap PEC cell with two electrodes featuring complementary light absorption.

In terms of the photoelectrodes' arrangement, a PEC system can be typically categorized into S2, D2, and D4 configurations (Fig. 3).^64^ The S2 configuration represents a single-bandgap PEC cell consisting of at least an anode and a cathode, with one electrode made from an n- or a p-type semiconductor capable of capturing sunlight. For example, when the photoanode is an n-type semiconductor, the cathode is typically a standard electrode material, such as Pt. When the photoanode absorbs light with sufficient energy, electrons in the VB are excited to the CB, leaving behind holes in the VB. Following charge separation, driven by either an internal or external electric field, the holes migrate to the electrode surface, facilitating an oxidation reaction, while the electrons travel through the external circuit to the counter electrode, where they drive a reduction reaction. To enable the desired reaction, the semiconductor in a single-bandgap PEC cell must meet specific criteria. Particularly, its bandgap must be larger than the thermodynamic requirement for the overall reaction (*e.g.*, >1.23 eV for water splitting), with its CB and VB appropriately positioned to straddle the reduction and oxidation potentials (*e.g.*, CB < 0 V *vs.* RHE for H_2_ evolution and VB > 1.23 V *vs.* RHE for O_2_ evolution or 0.003 V *vs.* RHE for glycerol oxidation).^65^ However, significant energy losses during charge separation, charge transport, and surface reaction kinetics typically result in overpotentials, necessitating a semiconductor with a higher bandgap (*e.g.*, >1.6 eV for water splitting).^66^ This requirement limits material choices, and photons with energy lower than the bandgap are practically not utilized.

To broaden the solar spectrum absorption and mitigate the constraints of a single semiconductor, PEC systems can incorporate two photoactive materials, one as the photoanode and the other as the photocathode, classified as D2 or D4 configurations. In the D2 configuration, the two photoelectrodes are illuminated side by side, whereas the D4 configuration employs a tandem structure, where the two electrodes are optically stacked to achieve complementary light absorption. Due to the increased illuminated area, the theoretical solar conversion efficiency of the D2 configuration is lower than that of the D4 configuration. In the D4 setup, the VB position of the photoanode must be more positive than the oxidation potential, while the CB position of the photocathode must be more negative than the reduction potential. The complementary absorption of light by both electrodes allows transmitted light with lower energy to be captured by the second photoelectrode, improving light utilization. This approach enables an ideal theoretical solar conversion efficiency of up to 27%, even when accounting for energy losses (0.8 eV per absorbed photon).^64^

### 2.3. The need for surface engineering in PEC biomass valorization

Biomass valorization often yields multiple products from a single feedstock, yet not all products are desirable. From an economic standpoint, high-value products, such as DHA and GLAD from glycerol oxidation or glycolic acid from ethylene glycol oxidation, are typically preferred.^67^ The formation of multiple products reflects inefficient energy utilization and increases the costs of purification and separation, raising overall production expenses. Additionally, unwanted byproduct intermediates can also occupy active reaction sites, hindering catalysis. Consequently, achieving high selectivity toward a single desired product is essential in PEC biomass conversion. Several strategies have been proposed to enhance selectivity, including electrolyte composition and feedstock concentration adjustment.^68–70^ However, since most reactions occur at the solid–liquid interface, the properties of the photoelectrode surface critically govern both reactivity and selectivity.

In PEC biomass valorization, surface engineering enhances conversion efficiency and selectivity through several key factors. One critical factor is surface area and morphology. Nanostructured surfaces, such as nanoporous or nanoarray architectures, generally outperform thin-film electrodes due to their increased active sites for biomass adsorption and subsequent reaction. Furthermore, nanostructuring is an effective strategy to shorten the transport length for photoexcited charges, reducing bulk recombination losses.^71,72^ Another key aspect involves surface defects and functionalization. The electronic state at the surface differs from that of the bulk due to abrupt termination at the interface. Surface defects and adsorbed functional species can significantly influence catalytic behavior by tuning the adsorption energy of reactants and products, a crucial parameter in catalysis according to the Sabatier principle.^73–75^ Moreover, surface functionalization can modulate charge transfer kinetics and recombination rates, directly affecting the overall reaction efficiency.^76,77^

The exposed facets on a material surface further dictate its catalytic performance. During crystal formation, facets with higher surface energies tend to grow rapidly and eventually diminish, while lower-energy facets remain stable.^78^ Uncontrolled growth thus often yields less reactive surfaces. Since different facets exhibit distinct atomic arrangements and electronic structures, they can enable unique reaction pathways in biomass conversion.^79^ For instance, some facets preferentially accumulate holes, while others favor electron accumulation,^80,81^ allowing selective tuning of the reaction environment. The combination of these specific facets can create built-in electric fields, enhancing charge separation.^82,83^

Finally, the local surface environment also plays a significant role. Properties such as hydrophilicity, hydrophobicity, and surface polarity impact the solubility, diffusion, and adsorption of biomass feedstocks. Tailoring surface wettability can improve interfacial interactions between the electrode, electrolyte, and biomass molecules, enhancing reaction efficiency.^59,84^ Furthermore, the polarizability of the surface has been reported to affect surface energy,^85^ band bending,^86^ and excited-state deactivation pathways,^87^ all of which influence PEC reaction dynamics. In some cases, local confinement effects can restrict the adsorption modes of biomass molecules, further refining reaction selectivity.

### 2.4. PEC biomass and CO_2_ valorization

Among the various PEC biomass valorization reactions, the glycerol oxidation reaction (GOR) is one of the most widely studied. Glycerol, a key by-product of biodiesel production, is abundant and inexpensive. These properties make it an ideal feedstock for conversion into value-added chemicals. Its three hydroxyl functional groups offer multiple oxidation pathways. For example, two-electron oxidation of the primary and secondary hydroxyl groups can yield glyceraldehyde (GLAD) and 1,3-dihydroxyacetone (DHA), respectively. Further oxidation can lead to more C_3_ products such as glyceric acid (GLYA) and lactic acid (LA). When C–C bond cleavage occurs, C_2_ and C_1_ products such as glycolic acid (GA) and formic acid (FA) can be formed. Among these, DHA and GLAD exhibit high market value, while formic acid has broad industrial demand, drawing significant interest to these transformations.^23–26^ In addition to glycerol, glucose (C_6_H_12_O_6_), the most abundant monosaccharide, has also been extensively studied in PEC systems due to its polyhydroxy structure, which allows diverse oxidation pathways. Oxidation of the C–OH groups produces C_6_ carboxylic acids such as gluconic acid and glucaric acid, while C–C bond cleavage can yield shorter-chain sugars and acids like arabinose, erythrose, glycolic acid, and formic acid.^88^ Conventional electrochemical or catalytic oxidation of glucose often requires noble metal catalysts, alkaline media, and O_2_.^89,90^ These conditions are transferable to PEC systems, though challenges persist, including overoxidation and uncontrolled C–C cleavage, which hinder selectivity. Such limitations call for surface engineering strategies, which will be discussed in the subsequent sections.

Another target in biomass valorization is the oxidation of 5-hydroxymethylfurfural (HMF) to 2,5-furandicarboxylic acid (FDCA). HMF, derived from cellulose and hemicellulose, is a key platform molecule in biorefineries.^91,92^ Its PEC oxidation to FDCA, a valuable monomer for bioplastics like polyethylene furanoate (PEF), has attracted growing attention.^93^ This reaction proceeds *via* sequential oxidation of the aldehyde and alcohol functional groups in HMF, forming intermediates such as 5-hydroxymethyl-2-furancarboxylic acid (HMFCA) and 5-formyl-2-furancarboxylic acid (FFCA). Achieving high selectivity to FDCA under PEC conditions requires careful control of active sites, surface potential, and light-assisted oxidation kinetics.^94^ Integration of co-catalysts and tuning of semiconductor surfaces are emerging strategies to promote FDCA formation while suppressing side reactions. In a similar context, aromatic alcohols such as benzyl alcohol can be selectively oxidized to benzaldehyde or further to benzoic acid under PEC conditions.^95,96^ These reactions represent valuable model systems for studying PEC oxidation mechanisms due to their relatively well-defined electron-transfer steps and stable intermediates. Oxidation of benzyl alcohol proceeds first to benzaldehyde, which can undergo further PEC oxidation to benzoic acid depending on the applied bias and reaction time. In addition, CO_2_ valorization has emerged as a parallel strategy for utilizing solar energy in a sustainable manner.^97^ The PEC reduction of CO_2_ to C_1_–C_3_ products such as CO, formic acid, methanol, ethanol, and acetic acid not only mitigates greenhouse gas emissions but also provides renewable chemical feedstocks. Surface engineering, including the incorporation of metal single atoms, plasmonic nanoparticles, or molecular catalysts, can steer reaction pathways, in particular towards multi-carbon products, improve CO_2_ adsorption, and enhance charge separation.^32^

Table 1 summarizes all the reactions above, which are the target reactions covered in this review. The table includes the reactants, products, reaction pathways, and, most importantly, their corresponding product utilizations.

**Table 1:** Representative biomass and CO_2_ valorization reactions

Feedstock	Product(s)	Reaction pathway(s)	Product utilization
Glycerol	Dihydroxyacetone	Oxidation^98^	Cosmetics, pharmaceuticals, fine chemicals, food^28,65,99^
		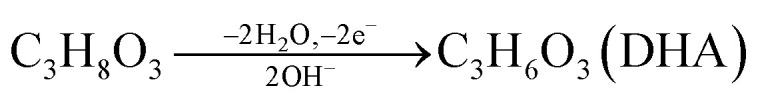	
	Formic acid	Oxidation^100^	Pharmaceuticals, agriculture, fuel technology, de-icing agents^101–103^
		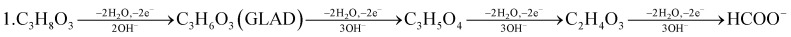	
		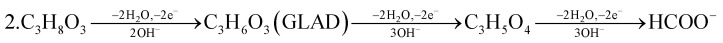	
	Glyceraldehyde	Oxidation^100^	Pharmaceuticals, food, cosmetics, fuel production^104,105^
		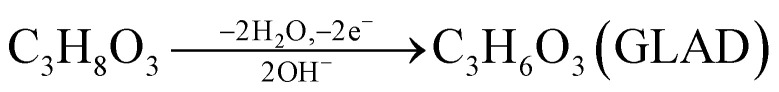	
	Lactic acid	Oxidation^106^	Pharmaceuticals, plastics, industrial cleaning, animal feed additives, food, personal care^107–111^
		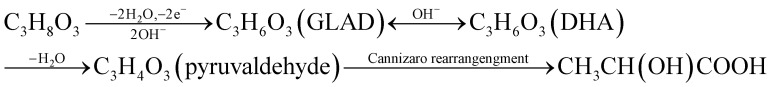	
Glucose	Glucaric acid	Oxidation^88^	
		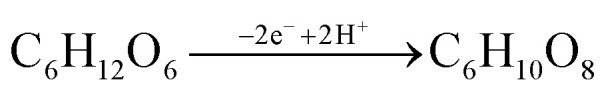	Pharmaceuticals, corrosion inhibitors, de-icing agents, food additives, detergents, textile industry, personal care^112–114^
Benzyl alcohol	Benzoic acid	Oxidation^95^	
		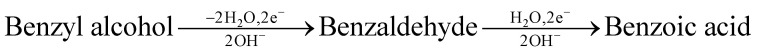	Food preservative, chemical industry, pharmaceuticals, industrial products^95,96^
	Benzaldehyde	Oxidation^96^	
		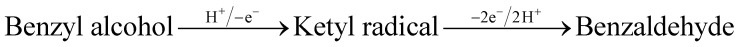	Coatings, textiles, food, pharmaceutical intermediates, agrochemicals, cosmetics^95,96^
HMF	FDCA	Oxidation^115^	
		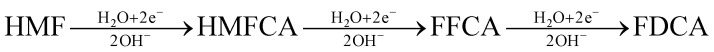	Fine chemicals^115^
CO_2_	CO	Reduction^97^	
		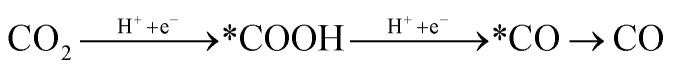	Metallurgical, semiconductors, graphene, chemical feedstock, food processing^116–118^
	Formic acid	Reduction^97^	
		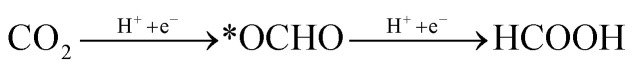	Pharmaceuticals, agriculture, batteries, de-icing agents^119,120^
	Formate	Reduction^121^	
	Acetic acid	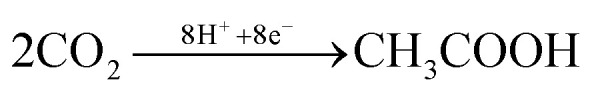	Chemical feedstock, pharmaceuticals, textiles, cosmetics^97,121,122^
	Methanol	Reduction^97^	
		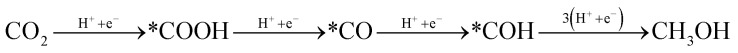	Chemical feedstock, biodiesel production, automotive fuel cells, construction, agriculture, water treatment^123–125^
	Methane	Reduction^97^	
		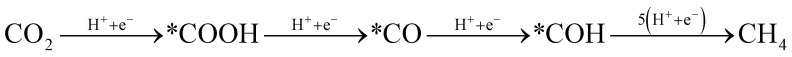	Fuel, automotive, aerospace, electronics^126–128^
	Ethylene	Reduction^97^	
		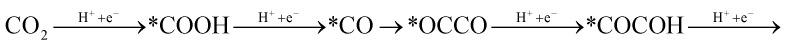	Plastics for packaging, construction, automotive, textiles, agriculture^129–131^
		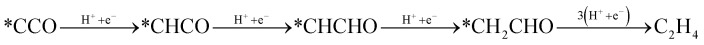	
	Ethanol	Reduction^97^	
		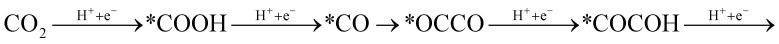	Bio-based solvents, pharmaceuticals, food, fuel^132,133^
		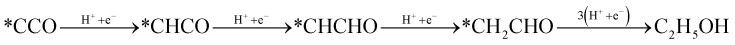	

## 3. Surface engineering for selectivity control in PEC biomass valorization

The surface properties of photoelectrodes play a fundamental role in determining the reactivity, efficiency, and selectivity of PEC biomass conversion. While optical and electronic modifications are commonly employed to regulate charge carrier dynamics, molecular adsorption is collectively governed by surface composition, wettability, and geometry. By precisely tuning these properties, it is possible to promote the preferential binding of target molecules while suppressing undesired competitive adsorption.^134–136^ In this section, we comprehensively discuss various surface engineering strategies, such as surface functionalization, crystal facet tuning, defect engineering, and nanostructuring, that enhance selectivity in PEC biomass and CO_2_ valorization. While examples specific to biomass valorization are available, they remain somewhat limited; we therefore also draw insights from PEC CO_2_ reduction systems, where a similar selectivity limitation exists and analogous surface engineering approaches (*e.g.*, active site control and interfacial modulation) have been more extensively studied. The discussed strategies offer precise control over active sites, charge transfer, and interactions with complex biomass feedstocks, enabling optimized reaction pathways for improved selectivity.

### 3.1. Surface functionalization

Surface functionalization plays a pivotal role in tailoring photoelectrode properties to enhance both the efficiency and selectivity of PEC biomass and CO_2_ valorization.^137–139^ This section delves into various surface engineering strategies, including the incorporation of nanoparticles, quantum dots, single-atom metals, layered double hydroxides, and molecular co-catalysts. Each approach offers unique advantages in optimizing light absorption, promoting charge separation, providing abundant active sites, and precisely tuning reaction pathways to achieve desired products. By decorating the surface of photoelectrodes, these strategies overcome limitations such as charge recombination and non-selective reactions, paving the way for more efficient and selective PEC conversion processes.

#### 3.1.1. Nanoparticles

Incorporating metal or metal compound nanoparticles (NPs) is a widely adopted strategy to utilize heterostructuring in PEC systems.^140^ One interesting phenomenon is localized surface plasmon resonance (LSPR), where incident light excites collective oscillations of conduction electrons in metal NPs.^141^ This unique property allows metal NPs to concentrate electromagnetic energy at their surfaces, generating “hot spots” that enhance light absorption and amplify localized electromagnetic fields.^142–145^ These effects significantly boost photocatalytic activity by increasing the number of photogenerated charge carriers available for PEC reactions. Additionally, the LSPR effect enables metal NPs to extend the system's light absorption spectrum into the visible and near-infrared regions, further improving PEC performance.^146–148^ Finally, the LSPR effect may also drive the selectivity of PEC biomass and CO_2_ valorization reactions through the modification of the energetic landscape induced by the enhanced electromagnetic field.^35,149^

**Fig. 4:**
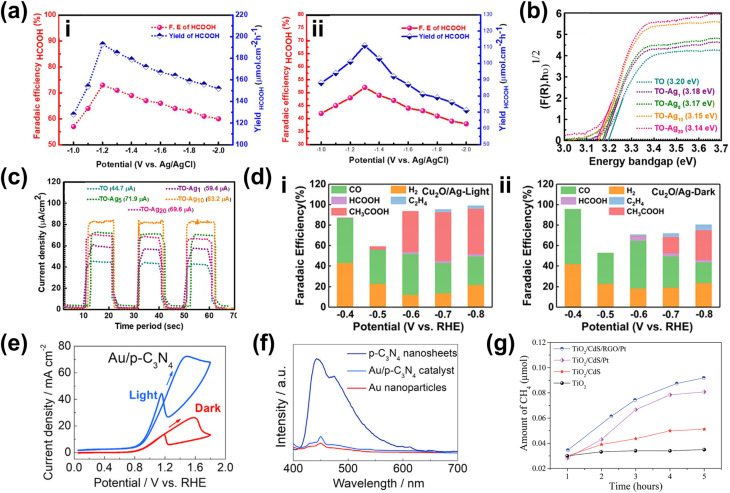
NPs in enhancing selectivity through light harvesting and charge transfer enhancement. (a) FE and yield of formate on TiO_2_–Ag NP photoelectrodes (i) with and (ii) without light illumination. (b) Tauc plot comparing the optical bandgap of bare TiO_2_ and Ag NP-decorated TiO_2_. (c) Photocurrent density of TiO_2_–Ag NP photoelectrodes with varying Ag NP concentrations. The photocurrent density shows an increasing trend up to 20 mM (TiO_2_–Ag_20_), beyond which a decline is observed despite a reduced bandgap. Adapted from ref. 154 with permission. Copyright 2022 The Korean Society of Industrial and Engineering Chemistry. (d) FE of products on the CuO_2_/Ag NP photoelectrode at different applied biases (i) with and (ii) without light illumination. Under illumination, multi-carbon products, acetic acid (CH_3_COOH, red) and ethylene (C_2_H_4_, blue), dominate. A lower potential is required to achieve the increase in selectivity when the reaction is conducted under light illumination. Adapted from ref. 154 with permission. Copyright 2022 The Royal Society of Chemistry. (e) Cyclic voltammetry of Au NPs/p-C_3_N_4_ photoelectrodes under dark and illuminated conditions. (f) Photoluminescence spectra of p-C_3_N_4_ nanosheets, the Au NPs/p-C_3_N_4_ catalyst, and Au NPs. The decrease in light emission around the 460 nm wavelength indicates that the interface electric field of the resulting Au NPs/p-C_3_N_4_ heterojunction effectively separates the photogenerated charge carriers. Adapted from ref. 158 with permission. Copyright 2021 Elsevier Inc. (g) Methane production from photocatalytic CO_2_ reduction in the presence of water on bare and modified TiO_2_ photoelectrodes. Reproduced from ref. 159 with permission. Copyright 2015 The Royal Society of Chemistry.

One strategy to leverage NPs for enhancing PEC biomass valorization selectivity is utilizing them as efficient light harvesters. By channeling additional energy into an already selective photoelectrode, metal NPs facilitate the generation of high-energy charge carriers, promoting improved PEC activity.^150^ This approach is commonly employed, often using simple deposition techniques such as drop-casting.^151,152^ For instance, Au_3_Cu NPs have been shown to enhance the PEC performance of CO_2_ reduction on Si nanowires, improving the faradaic efficiency (FE) for CO to nearly 80% under illumination.^153^ Another example involves loading Ag NPs onto TiO_2_, leading to an increase in the FE of CO_2_-to-formic acid (FA) conversion from 52% in the absence of illumination to 73% under light exposure (Fig. 4a).^154^ This increase in FE is analogous to the enhanced current density and light absorption, indicating the effectiveness of the deposited NPs in improving charge transfer and acting as a light harvester. However, optimizing the deposited NP concentration is crucial in this strategy. Varying the Ag NP loading on TiO_2_ results in distinct absorption bands (Fig. 4b), indicating changes in the system's (apparent) bandgap.^155^ While increased NP concentration reduces the bandgap and enhances charge transfer capability, excessive deposition can lead to agglomeration, reducing the active surface area and negatively impacting performance (Fig. 4c).^156^ A similar enhancement under illumination is observed when Ag NPs are deposited onto Cu_2_O nanowires using vacuum thermal evaporation.^157^ This method improves CO_2_ reduction selectivity and increases the overall yields of multi-carbon products (Fig. 4d).

Beyond enhancing light absorption, another strategy for leveraging NPs in PEC systems involves their role in promoting charge separation, by acting as electron reservoirs or sinks. This function is critical in reducing charge recombination losses and ensuring selective transfer of photogenerated carriers to targeted reaction sites. For instance, PEC glycerol oxidation on polymeric C_3_N_4_ (p-C_3_N_4_) nanosheets exhibits a significant performance enhancement upon incorporating Au NPs.^158^ In this case, the interaction between Au NPs and p-C_3_N_4_ suppresses charge carrier recombination while promoting the accumulation of photogenerated holes on the NPs. These synergistic effects result in a higher current density (Fig. 4e) and a suppressed photoluminescence signal around 460 nm (Fig. 4f). A promising extension to this strategy involves employing multiple types of NPs. For example, TiO_2_/CdS and Pt NPs have been integrated onto reduced graphene oxide (rGO) to enhance the CO_2_-to-methane yield.^159^ Here, TiO_2_ predominantly absorbs in the UV region, limiting the PEC performance. However, this limitation is mitigated by incorporating CdS, which extends the light absorption into the visible range. Moreover, introduction of Pt NPs further enhances methane production (Fig. 4g), attributed to the synergistic effect of the NPs and rGO in photogeneration, charge separation, and carrier transport. Upon exposure to visible light, electron–hole pairs are generated in both semiconductors. Due to the specific band alignment at the TiO_2_/CdS interface, photogenerated electrons transfer to the CB of TiO_2_, while holes remain in the VB of CdS. This spatial separation of charge carriers reduces recombination. Additionally, rGO, a well-known electron-accepting material with excellent conductivity due to its two-dimensional planar structure, facilitates rapid charge transfer. This efficient transport of charge carriers across the graphene sheets further enhances charge separation, ultimately suppressing the electron–hole recombination.

**Fig. 5:**
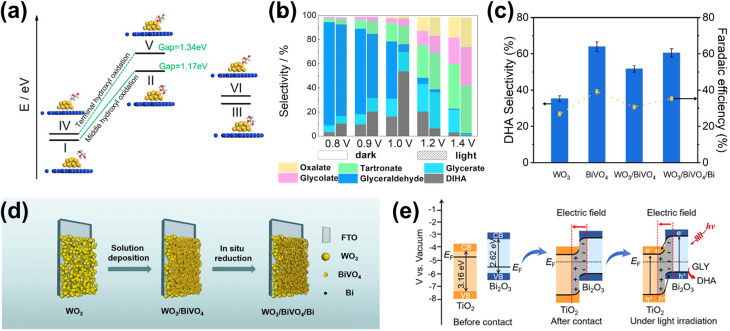
NP-assisted selectivity tuning in PEC glycerol oxidation. (a) Potential energy for the oxidation of terminal and middle hydroxyls of glycerol on the Au NPs/p-C_3_N_4_ photoelectrode. The adsorption energies of middle and terminal hydroxyls are calculated to be −1.42 eV and −1.27 eV, respectively, which are higher than that of pristine p-C_3_N_4_. The theoretical calculations of the oxidation potential energy and adsorption energy are consistent with the heightened selectivity of the Au NPs/p-C_3_N_4_ photoelectrode towards DHA. (b) Product selectivity for glycerol oxidation on Au NPs/p-C_3_N_4_ at various potentials with and without light illumination. The maximum selectivity of DHA (gray) is achieved at 1.0 V_RHE_ potential under light illumination. Adapted from ref. 158 with permission. Copyright 2021 Elsevier Inc. (c) DHA selectivity and FE on pristine WO_3_, BiVO_4_, and modified photoelectrodes. The reaction is conducted under light illumination and 1.2 V_RHE_ applied bias in 0.5 M Na_2_SO_4_ with glycerol. Adapted from ref. 163 with permission. Copyright 2023 The Royal Society of Chemistry. (d) Fabrication steps of the WO_3_/BiVO_4_/Bi NP photoanode. (e) Energy band diagram of Bi_2_O_3_ and TiO_2_, along with the proposed mechanism for PEC glycerol oxidation in the Bi_2_O_3_/TiO_2_ p–n junction under illumination. Adapted from ref. 164 with permission. Copyright 2022 American Chemical Society.

In terms of selectivity control, NPs can be employed to promote specific reaction pathways. The enhanced electromagnetic field near the plasmonic surface lowers activation energy for certain reactions, selectively stabilizing key intermediates or transition states.^35,149^ Additionally, plasmonic “hot spots” enhance the adsorption of biomass molecules near active sites, improving surface interactions and reaction kinetics.^160–162^ By selectively strengthening the interaction energy between the plasmonic surface and specific functional groups, these NPs drive targeted reactions with high precision. In this context, the impact of NPs on the selectivity of PEC reactions has been well demonstrated. For instance, in PEC glycerol oxidation, the incorporation of Au NPs on p-C_3_N_4_ enhances selectivity toward dihydroxyacetone (DHA).^158^ In this approach, a chemical reduction method was used to evenly distribute the NPs on the C_3_N_4_ photoelectrode. Calculations reveal that the potential energy for middle hydroxyl oxidation is higher than that for the terminal hydroxyl (Fig. 5a), resulting in selective dehydrogenation and oxidation of glycerol to DHA with a selectivity of up to 53.7% (Fig. 5b). Beyond Au NP-modified systems, Bi-based NPs have also shown remarkable potential in tuning PEC glycerol oxidation selectivity, particularly toward DHA.^163^ A notable example involves the *in situ* reduction of WO_3_/BiVO_4_ using hydrazine hydrate to produce a WO_3_/BiVO_4_/Bi NP photoanode, which achieves a DHA selectivity of up to 60.6%, surpassing the 53.3% observed for bare WO_3_/BiVO_4_ (Fig. 5c). This process reduces Bi^3+^ in BiVO_4_ to metallic Bi^0^, forming Bi^0^/Bi^3+^ active sites (Fig. 5d). The hydrazine reduction is carefully controlled to prevent over-reduction, which could compromise the structural integrity of the composite. The improved selectivity is attributed to the enhanced affinity of the BiVO_4_/Bi NP surface for the middle hydroxyl of glycerol, with the NPs further promoting adsorption. DFT calculations confirm that glycerol's terminal and middle hydroxyl groups (*i.e.*, primary and secondary –OH, respectively) preferentially adsorb onto Bi^0^ sites at the Bi^0^/Bi^3+^ interface. The rate-limiting step in this process is carbon-centered radical formation *via* photogenerated hole-driven dehydrogenation. The activation barrier for middle-carbon radical formation is lower than that for terminal radicals, resulting in the mechanism that favors DHA production. A similar trend is observed in Bi_2_O_3_ NP-modified TiO_2_ photoanodes. In this system, Bi is first electrodeposited onto TiO_2_, followed by electro-oxidation to form a Bi_2_O_3_/TiO_2_ heterojunction (Fig. 5e).^164^ This structure achieves an impressive glycerol-to-DHA selectivity of 75.4%. The Bi_2_O_3_ NPs exhibit low DHA adsorption capability, preventing further oxidation and contributing to high selectivity. Additionally, the Bi_2_O_3_/TiO_2_ heterojunction has enhanced charge transfer and photogenerated hole utilization due to the formation of a p–n junction. The important role of the Bi_2_O_3_ NPs in tuning selectivity is evident from the fact that bare TiO_2_ favors FA formation instead; although FA is another high-value glycerol derivative, its market price is far below that of DHA.

**Fig. 6:**
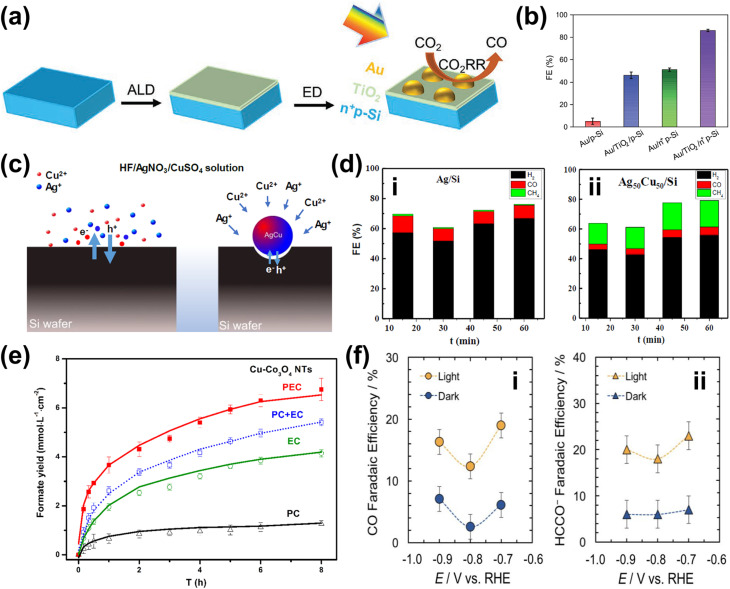
NP-enabled selectivity tuning in PEC CO_2_ reduction toward single-carbon products and other biomass valorization reactions. (a) Fabrication process of the Au NPs/TiO_2_/n^+^p-Si photoelectrode. (b) FE for CO at −0.4 V_RHE_ on Au NP-modified p-Si photoelectrodes. Adapted from ref. 166 with permission. Copyright 2022 Wiley-VCH GmbH. (c) Schematic illustration of AgCu NP formation and deposition on a Si wafer *via* metal-assisted chemical etching. Ag^+^ and Cu^2+^ ions are spontaneously reduced upon contact with the Si surface, extracting electrons from the Si valence band and forming Ag^0^ and Cu^0^ nuclei. HF solution facilitates Si dissolution, creating a porous structure that supports ion reduction and NP growth. (d) FE of PEC CO_2_ reduction products on (i) Ag and (ii) AgCu NP-modified Si photoelectrodes. Adapted from ref. 167 with permission. Copyright 2023 American Chemical Society. (e) Performance of Cu NPs on the Co_3_O_4_ NT photoelectrode for selective CO_2_ reduction to formate. Adapted from ref. 169 with permission. Copyright 2015 American Chemical Society. (f) Performance of Cu NPs on the p-NiO film to selectively reduce CO_2_ to (i) CO and (ii) formate. Adapted from ref. 170 with permission. Copyright 2020 American Chemical Society.

Beyond glycerol oxidation, selectivity tuning through the incorporation of NPs has also been demonstrated for PEC CO_2_ reduction. Similar to glycerol oxidation, NPs can serve as adsorption centers for CO_2_ molecules, selectively adsorbing key intermediates that directly influence reaction selectivity—particularly toward single-carbon products.^165^ To tune the reaction selectivity toward CO, the system must be engineered to promote CO_2_ activation into *COOH and facilitate *CO desorption. In this regard, employing Au NPs^166^ and AgCu bimetallic NPs^167,168^ has proven to be highly effective for achieving high selectivity in the CO_2_ reduction reaction toward CO. For the former, electrodeposited Au NPs exhibit a synergistic effect with TiO_2_ (Fig. 6a), jointly enhancing CO_2_ adsorption and promoting the kinetics of CO production by accelerating both the *COOH formation step and the subsequent desorption of *CO from active sites. This results in an improved FE for CO, reaching 86% (Fig. 6b). Alternatively, the use of AgCu NPs offers a unique tunability mechanism based on composition modulation. The AgCu NPs are embedded onto a p-Si film *via* immersion in an HF solution containing Ag^+^ and Cu^2+^ ions (Fig. 6c). The reaction is more selective towards CO when Ag is the dominant component, and the selectivity shifts to methane when Cu is introduced (Fig. 6d(i and ii)). This tunability through the modulation of Ag and Cu ratio in the NPs arises from the electronic and geometrical effects generated by the nanoscale coupling of the two metals. On the one hand, Ag is responsible for the reduction of CO_2_ to CO and on the other, Cu continues the reduction process to single or multi-carbon. It is also important to highlight that the geometric structure plays a crucial role in tuning the reaction selectivity. In this case, the selectivity towards the single-carbon product, methane, rather than multi-carbon products, is possible due to the presence of intermixed Ag and Cu domains, which preferentially produce methane. Additionally, similar selectivity modulation is observed when NPs are embedded onto p-Si micropillars.

Formate is another valuable single-carbon product of PEC CO_2_ reduction. To tune the reaction toward formate, it is essential to stabilize *OCHO and *HCOOH intermediates while reducing the desorption energy of *HCOOH. Cu-based NPs are particularly effective for this purpose.^169,170^ In this process, chemisorbed CO_2_ attracts protons from the electrolyte, forming a chemisorbed HCOO radical *via* a stable *OCHO intermediate. Electron transfer to this radical species leads to formate desorption. Additionally, CO_2_ radicals at Cu active sites can react with surface-bound H atoms to generate formate. This strategy has been successfully demonstrated through the integration of Cu NPs into Co_3_O_4_ nanotube arrays and p-NiO films. In the first case, Cu NPs are electrodeposited onto nanotube arrays, yielding 6.75 mmol L^−1^ cm^−2^ of formate over 8 hours of reaction with nearly 100% selectivity (Fig. 6e). In the second case, a 3 nm Cu layer is deposited onto a p-NiO film *via* electron-beam physical vapor deposition, resulting in a formate FE of at least 20% (Fig. 6f(i and ii)). Notably, bare p-NiO exclusively produces H_2_ with an FE of 98%, underscoring the crucial role of Cu NPs in selectively tuning the reaction toward formate.

**Fig. 7:**
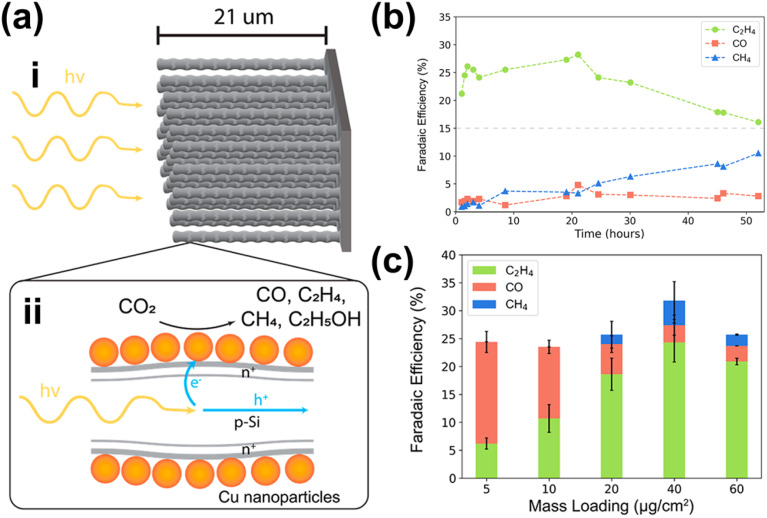
NP-enabled selectivity tuning in PEC CO_2_ reduction toward multi-carbon products. (a) Cu NPs/Si NW photocathode: (i) illustration and (ii) PEC reaction mechanism. (b) PEC CO_2_ reduction performance of the Cu NPs/Si NW photocathode over 50 h. (c) FE of PEC CO_2_ reduction products as a function of Cu NP loading. Adapted from ref. 171 with permission. Copyright 2022 American Chemical Society.

For PEC CO_2_ reduction to multi-carbon products, the corresponding selectivity largely depends on the specific ability of the metal NPs to facilitate carbon–carbon (C–C) coupling. Notably, Cu is the only known metal thus far capable of catalyzing this crucial step. One example of utilizing Cu NPs for this purpose involves incorporating them into Si nanowires (Fig. 7a(i and ii)) *via* a drop-casting method.^171^ This approach enables the selective conversion of CO_2_ to ethylene for up to 50 hours while maintaining a FE above 15% (Fig. 7b). Moreover, increasing the Cu loading further enhances this selectivity (Fig. 7c). However, excessive Cu deposition leads to NP accumulation at the base of the nanowire arrays, which leads to agglomeration and decreased active surface area,^172^ negatively impacting the conversion performance. Additionally, AuCu bimetallic NPs can be integrated with photoelectrodes to achieve selective PEC CO_2_ reduction to ethanol.^173^ While Cu facilitates C–C coupling, Au plays a critical role in modifying the binding energies of key reaction intermediates, such as *CH_2_CHO, *CH_3_CHO, and *CH_3_CH_2_O, thereby contributing to the reaction selectivity. However, to the best of our knowledge, this strategy has only been demonstrated in electrocatalysis, where the NPs are deposited on Cu submicrocone arrays (Cu SCAs) *via* an *in situ* reduction method. Nevertheless, this approach presents a promising pathway for achieving selective PEC CO_2_ reduction to multi-carbon products.

In conclusion, the examples discussed above highlight the ability of NPs to enhance and tune PEC biomass and CO_2_ valorization selectivity. In particular, they concentrate electromagnetic energy on their surfaces, generating “hot spots” that enhance light absorption and amplify localized electromagnetic fields, in turn boosting photocatalytic activity by increasing photogenerated charge carriers available for PEC reactions. Additionally, NPs extend the system's light absorption spectrum into the visible and near-infrared regions, further improving PEC performance. They also act as efficient light harvesters, channeling additional energy into selective photoelectrodes to promote improved PEC activity. Beyond light absorption, NPs promote charge separation by acting as electron reservoirs or sinks, which is critical in reducing charge recombination losses and ensuring selective transfer of photogenerated carriers to targeted reaction sites. In the context of selectivity tuning, the enhanced electromagnetic field near the plasmonic surface lowers activation energy for certain reactions, selectively stabilizing key intermediates or transition states. NPs also enhance the adsorption of biomass molecules near active sites, improving surface interactions and reaction kinetics. Given their versatility, decorating photoelectrodes' surface with NP cocatalysts holds significant potential for selectivity tuning and enhancement in a broad range of PEC reactions, including methane oxidation,^174,175^ ethanol oxidation,^176^ and nitrate reduction.^177^ However, a crucial consideration with NP surface functionalization relates to its concentration optimization, as excessive deposition can lead to agglomeration, which subsequently reduces the active surface area and negatively impacts performance. A summary of the role of NPs in enhancing and tuning PEC reaction selectivity is presented in Table 2.

**Table 2:** Summary of nanoparticle (NP) utilization on the surface of photoelectrodes for PEC biomass valorization selectivity enhancement and tuning applications

Photoelectrode^a^	NP role	Deposition method	Reaction	Target product(s)	Selectivity	FE	Reaction conditions	Ref.
Au_3_Cu NPs/Si NW	Light harvester	Drop-casting	CO_2_ reduction	CO	N/A	80%	• Electrolyte: CO_2_-saturated KHCO_3_ (0.1 M, pH 6.8)	153
							• Applied bias: −0.2 V_RHE_	
							• Light: AM 1.5G (100 mW cm^−2^)	
TiO_2_–Ag NPs	Light absorption and charge transport promoter	Immersion and calcination	CO2 reduction	FA	N/A	73%	• Electrolyte: CO_2_-saturated K_2_SO_4_ (0.5 M)	154
							• Applied bias: −1.2 V_Ag/AgCl_	
							• Light: 300 W Xe lamp	
Cu_2_O NW/Ag NPs	Light harvester and charge separation promoter	Vacuum thermal evaporation	CO_2_ reduction	Acetic acid	N/A	47.7%	• Electrolyte: CO_2_-saturated KHCO_3_ (0.1 M)	157
							• Applied bias: −0.7 V_RHE_	
							• Light: AM 1.5G (100 mW cm^−2^)	
Au NPs/C_3_N_4_	Promoting middle hydroxyl oxidation, charge transfer and separation promoter, light harvester	Immersion	Glycerol oxidation	DHA	53.7%	N/A	• Electrolyte: KOH (1 M) with glycerol (1 M)	158
							• Applied bias: 1.0 V_RHE_	
							• Light: AM 1.5G	
WO_3_/BiVO_4_/Bi NPs	Middle hydroxyl adsorption site, light harvester, charge transport promoter	*In situ* reduction	Glycerol oxidation	DHA	60.6%	N/A	• Electrolyte: Na_2_SO_4_ (0.5 M, pH 2) with glycerol (0.1 M)	163
							• Applied bias: 1.2 V_RHE_	
							• Light: AM 1.5G (100 mW cm^−2^)	
Bi_2_O_3_ NPs/TiO_2_ NRAs	Middle hydroxyl adsorption site and charge transfer promoter	Electrodeposition and electro-oxidation	Glycerol oxidation	DHA	75.4%	N/A	• Electrolyte: Na_2_SO_4_ (0.5 M, pH 2)	164
							• Applied bias: 1.0 V	
							• Light: AM 1.5G (100 mW cm^−2^)	
TiO_2_ NTs/CuO NPs	CO_2_ adsorption site, charge transport and separation promoter	Dip-coating	CO_2_ reduction	Methanol	N/A	57%	• Electrolyte: K_2_SO_4_ (0.1 M, pH 8)	165
							• Applied bias: 0.2 V_Ag/AgCl_	
							• Light: UV-visible	
Au NPs/TiO_2_/n^+^p-Si	Light harvester, charge separation and transport promoter, promoting COOH* formation and CO desorption	Electrodeposition	CO_2_ reduction	CO	N/A	86%	• Electrolyte: CO_2_-saturated KHCO_3_ (0.1 M)	166
							• Applied bias: −0.4 V_RHE_	
							• Light: AM 1.5G (100 mW cm^−2^)	
Ag NPs/p-Si	Light harvester, reaction and adsorption center	Metal-assisted chemical etching	CO_2_ reduction	CO	N/A	10%	• Electrolyte: CO_2_-saturated NaHCO_3_ (0.5 M)	167
							• Applied bias: −0.87 V_RHE_	
							• Light: AM 1.5G (100 mW cm^−2^)	
AgCu NPs/p-Si	Light harvester, reaction and adsorption center	Metal-assisted chemical etching	CO_2_ reduction	Methane	N/A	18.2%	• Electrolyte: CO_2_-saturated NaHCO_3_ (0.5 M)	167
							• Applied bias: −0.87 V_RHE_	
							• Light: AM 1.5G (100 mW cm^−2^)	
Ag NPs/Si MPs	Light harvester, reaction and adsorption center	Metal-assisted chemical etching	CO_2_ reduction	CO	N/A	12.1%	• Electrolyte: CO_2_-saturated NaHCO_3_ (0.5 M)	168
							• Applied bias: −0.87 V_RHE_	
							• Light: AM 1.5G (100 mW cm^−2^)	
AgCu NPs/Si MPs	Light harvester, reaction and adsorption center	Metal-assisted chemical etching	CO_2_ reduction	CO and methane	N/A	16.7% (CO) and 9% (methane)	• Electrolyte: CO_2_-saturated NaHCO_3_ (0.5 M)	168
							• Applied bias: −0.87 V_RHE_	
							• Light: AM 1.5G (100 mW cm^−2^)	
Cu NPs–Co_3_O_4_ NTs	CO_2_ adsorption and formate desorption sites	Electrodeposition	CO_2_ reduction	Formate	100%	N/A	• Electrolyte: CO_2_-saturated Na_2_SO_4_ (0.1 M)	169
							• Applied bias: −0.9 V_SCE_	
							• Light: 10 mW cm^−2^	
Cu NPs/p-NiO	CO_2_ adsorption and activation to CO_2_^−^ sites	Electron-beam physical vapor deposition	CO_2_ reduction	Formate	N/A	20%	• Electrolyte: CO_2_-saturated K_2_CO_3_ (0.05 M)	170
							• Applied bias: −0.7 V_RHE_	
							• Light: 160 mW cm^−2^	
Cu NPs/Si NWs	C–C coupling catalyst	Drop-casting	CO_2_ reduction	Ethylene	N/A	25%	• Electrolyte: CO_2_-saturated KHCO_3_ (0.1 M)	171
							• Applied bias: −0.5 V_RHE_	
							• Light: AM 1.5G (100 mW cm^−2^)	
AuCu NPs/Cu SCAs	C–C coupling catalyst and CH_2_CHO*, CH_3_CHO*, and CH_3_CH_2_O* intermediate adsorption sites	*In situ* reduction	CO_2_ reduction	Ethanol and ethylene	N/A	29% (ethanol) and 16% (ethylene)	• Electrolyte: CO_2_-saturated KHCO_3_ (0.5 M)	173
							• Applied bias: −1.0 V_RHE_ (ethanol) and −1.1 V_RHE_ (ethylene)	
							• Light: no information	
TiO_2_/MOF(Ti)/Au NPs	Increasing surface roughness and light harvester	Photochemical	Methane oxidation	Methanol	N/A	65.32%	• Electrolyte: Na_2_SO_4_ (0.1 M)	174
							• Applied bias: 0.5 V_RHE_	
							• Light: AM 1.5G (100 mW cm^−2^)	

a MP: micropillar, NP: nanoparticle, NRA: nanorod array, NT: nanotube, NW: nanowire, and SCA: submicrocone array.

#### 3.1.2. Quantum dots

Heterostructuring with quantum dots (QDs) offers a transformative approach to increasing both conversion efficiency and selectivity in PEC of biomass. QDs are known for their size-tunable bandgap and high surface area-to-volume ratio, which could provide unique electronic and optical properties to influence reaction pathways.^178^ Distributing QDs onto photoelectrode surfaces often results in the introduction of additional energy levels in the heterostructure.^20,179,180^ These energy levels may be better aligned with the redox potentials of substrate molecules and/or biomass-derived intermediates. Additionally, QDs exhibit strong light absorption across a wide light spectrum.^181–183^ Hence, its presence can also boost the light harvesting ability of the overall photoelectrodes and improve conversion efficiency. Due to their small size, infiltrating and decorating the often-intricate photoelectrode morphologies (*e.g.*, nanostructured or porous) are possible. QDs can also function as co-catalysts in PEC biomass valorization.^37,180,184^ In this case, QDs can facilitate specific reaction pathways by acting as active sites for the adsorption and activation of reactants or intermediates. Their tunable surface properties and ability to generate a high density of reactive sites also enable precise modulation of the reaction environments.^185,186^ Moreover, their strong quantum confinement effect can create highly reactive electronic states that selectively interact with specific functional groups in complex biomass molecules.^187,188^ By serving as co-catalysts, QDs not only enhance the photocatalytic efficiency of PEC systems but also ensure a higher degree of control over product distribution, making them an invaluable component in the selective valorization of biomass. Another impact is enhanced selectivity through the QDs' ability to generate localized surface states that interact preferentially with certain intermediates.^37,189–191^ Last, the introduction of QDs can lead to direct photogenerated electrons or holes toward specific reaction sites by modulating the local electronic environment of the photoelectrodes.^192,193^ This effect ensures that the energy from charge carriers is channeled efficiently into selective reaction pathways, thereby reducing side reactions.

**Fig. 8:**
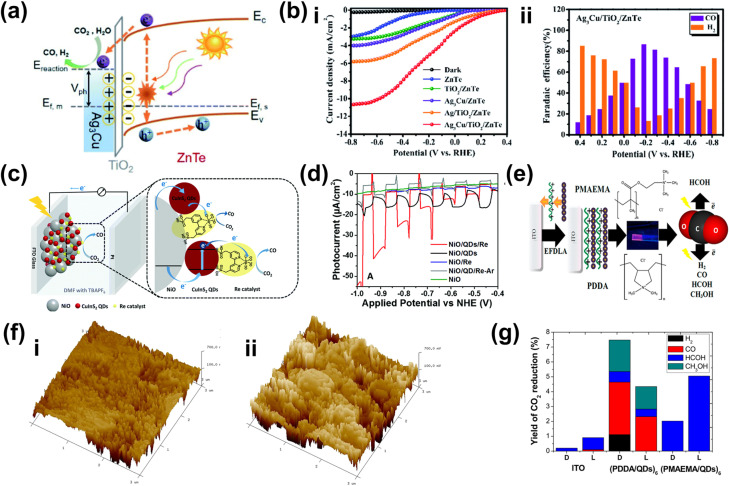
QDs as a co-catalyst for improved selectivity of the PEC CO_2_ reduction reaction. (a) Band diagram of the Ag_3_Cu/TiO_2_/ZnTe QD photocathode. The Ag_3_Cu NCs serve as a metallic collector on a TiO_2_-coated ZnTe QD semiconductor film. The diagram highlights key energy levels, including the conduction band (*E*_0_), valence band (*E*_V_), Fermi level of ZnTe (*E*_f,s_), Fermi level of the metal (*E*_f,m_), and the potential values of relevant chemical reactions (*E*_reaction_), providing insight into the charge transfer mechanism and photocatalytic activity of the system. (b) PEC CO_2_ reduction performance of the Ag_3_Cu/TiO_2_/ZnTe QD photocathode shown as (i) the comparison of photocurrent density and (ii) the FE of CO and H_2_ production on the photocathode as a function of the applied bias. Adapted from ref. 197 with permission. Copyright 2021 The Royal Society of Chemistry. (c) The schematic representation of a PEC cell designed for CO_2_ reduction featuring a NiO photocathode co-modified with CuInS_2_ QDs and a Re catalyst. (d) Linear-sweep voltammetry curves of the NiO photocathode recorded for various samples under chopped light illumination. Adapted from ref. 199 with permission. Copyright 2019 The Royal Society of Chemistry. (e) Schematic diagram of the electric field-directed layer-by-layer assembly on an ITO substrate under an applied potential of 1000 mV. This process involves the deposition of strong polycations with distinct structural differences—cyclic *versus* branched monomer structures—which influence the assembly mechanism. These structural variations can impact key factors such as the growth dynamics and adsorption kinetics of the multilayers, ultimately affecting the overall stability and performance of the assembled film. (f) 3-Dimensional AFM phase imaging of (i) ITO-(PDDA/QDs)_6_ and (ii) ITO-(PMAEMA/QDs)_6_ surfaces. (g) CO_2_ reduction product yields obtained by potential controlled electrolysis under dark conditions (D) and light irradiation (L) over 3 h with −450 mV applied bias. Adapted from ref. 200 with permission. Copyright 2015 American Chemical Society.

A common application for surface engineering with QDs found in the literature is in PEC CO_2_ reduction systems. In such a case, integrating QDs to form a metal-insulator-semiconductor (MIS) structure offers a promising strategy by improving charge transfer and providing abundant active sites, thereby enhancing catalytic activity and selectivity for the PEC reaction.^194–196^ One example is the Ag_3_Cu/TiO_2_/ZnTe QD structure for the CO_2_ reduction reaction to CO.^197^ Through this design, the MIS structure effectively couples interfacial charge transport, surface reaction processes, and long-term stability, offering a robust PEC photocathode design. Under light irradiation, the ZnTe QDs absorb photons, generating minority electrons that tunnel through the TiO_2_ layer to the Ag_3_Cu nanocrystals (NCs), where reduction reactions involving CO_2_ or H^+^ occur (Fig. 8a). Furthermore, the ultrathin TiO_2_ layer reduces surface defects on the ZnTe film and minimizes charge recombination. Overall, this strategy leads to significant performance enhancements, as demonstrated by the Ag_3_Cu/TiO_2_/ZnTe photocathode achieving a photocurrent density of −10.68 mA cm^−2^ at −0.8 V_RHE_ (Fig. 8b (i)) and a maximum FE of 86.5% for CO at −0.2 V_RHE_ (Fig. 8b(ii)). However, despite its effectiveness, this approach is often associated with high costs.^198^ As a cost-effective alternative, immersion techniques can instead be employed. One example utilizes CuInS_2_ QDs along with Re catalysts to serve as photosensitizers on NiO photocathodes for selective PEC CO_2_ reduction to CO (Fig. 8c).^199^ Specifically in this approach, the CuInS_2_ QDs were directly synthesized from an aqueous solution using l-cysteine immobilized on a 1 mm thick NiO electrode by immersion. This method yields small molecular sizes, allowing more penetration into mesoporous NiO films and providing more surface coverage. CuInS_2_ QDs, with a valence band (VB) positioned at 0.5 V and a conduction band (CB) at −1.3 V_NHE_, can thermodynamically transfer photogenerated holes into the VB of NiO, allowing efficient charge separation and reaction progression. Additionally, the CB of CuInS_2_ facilitates efficient electron transfer to the Re catalysts due to the more negative reductive potential of the QDs. A PEC CO_2_ reduction test shows that the deposited CuInS_2_ QDs significantly enhance the photocurrent, as shown in Fig. 8d, albeit with a relatively low CO_2_ to CO FE of 32% at −0.87 V_NHE_. Nevertheless, these examples demonstrate the versatility and capability of QDs as co-catalysts to enhance the selectivity of PEC CO_2_ reduction to CO.

Adding to the versatility of QD utilization for the PEC CO_2_ reduction reaction, incorporating QDs onto electrode surfaces through bondage with polymers or biomolecules unveils opportunities to tune the selectivity of the reaction. In particular, electrostatic assembly can be achieved using an electric field-driven layer-by-layer deposition of polycations, such as poly(diallyldimethylammonium) (PDDA) and poly(2-trimethylammonium)ethyl methacrylate (PMAEMA) (Fig. 8e).^200^ It is later revealed that the former sample exhibits a higher structural profile (Fig. 8f (i)), whereas the latter displays higher average roughness (Fig. 8f(ii)). This increased roughness is likely associated with a greater presence of microdomain structures due to the incorporation of the PMAEMA polycation. The polycations influence the assembly process and interact with the QDs, resulting in a subtle shift in the onset potential for both electrodes. These shifts enhance intermolecular charge transfer and improve electrocatalytic performance. The photoelectrocatalytic activity of the (PDDA/QDs)_6_ assembly yielded carbon monoxide, methanol, and smaller amounts of formaldehyde, whereas the (PMAEMA/QDs)_6_ assembly exclusively produced formaldehyde, emphasizing its selectivity for HCOH formation (Fig. 8g). The distinct reaction products suggest that variations in the surface structure and the resulting interactions between polycations, QDs, and CO_2_ molecules drive two distinct reduction pathways, the formaldehyde and carbene pathways.

Based on the discussion above, surface functionalization with QDs serves as a powerful platform for PEC biomass and CO_2_ valorization selectivity enhancement and tuning. Their distribution onto photoelectrode surfaces often introduces additional energy levels in the heterostructure, which may more suitably align with the redox potentials of substrate molecules and/or biomass-derived intermediates. QDs also exhibit strong light absorption across a wide spectrum, boosting the overall photoelectrode's light harvesting ability and improving conversion efficiency. Due to their small sizes, QDs can infiltrate and decorate intricate photoelectrode morphologies like nanostructured or porous designs. Their tunable surface properties and ability to generate a high density of reactive sites enable precise modulation of reaction environments. Moreover, their strong quantum confinement effect can create highly reactive electronic states that selectively interact with specific functional groups in complex biomass molecules. In terms of selectivity, QDs are able to generate localized surface states that interact preferentially with certain intermediates. Lastly, the introduction of QDs can direct photogenerated electrons or holes toward specific reaction sites by modulating the local electronic environment of the photoelectrodes, ensuring efficient channeling of energy into selective reaction pathways and reducing side reactions. However, several challenges remain for the practical deployment of QD-functionalized PEC systems. In colloidal QD-based photoelectrodes, interfacial charge-carrier recombination, limited catalytic selectivity, and poor operational stability in aqueous electrolytes continue to hinder PEC performance.^201^ Furthermore, carbon-based QD-sensitized semiconductor systems, in particular, suffer from low efficiency due to small surface area and high energy barriers for charge transport.^202^ In the case of CO_2_ reduction, systems employing QDs often favor water reduction in carbonated solutions, as its pathway is kinetically more accessible.^203^ Despite these limitations, the unique tunability, strong light absorption, and capacity for selective interfacial interactions make QDs a highly promising class of surface modifiers for advancing PEC biomass and CO_2_ valorization. The selected utilization of various QDs for PEC biomass and CO_2_ valorization and their respective performances are summarized in Table 3.

**Table 3:** Summary of quantum dot (QD) utilization on the surface of photoelectrodes for PEC biomass valorization selectivity enhancement and tuning applications

Photoelectrode	QD role	Deposition method	Reaction	Target product(s)	Selectivity	FE	Reaction conditions	Ref.
Ag_3_Cu/TiO_2_/ZnTe QDs	Charge separation and transport promoter, light harvester	Spin-coating	CO_2_ reduction	CO	6.8 CO : H_2_ ratio	N/A	• Electrolyte: CO_2_-saturated KHCO_3_ (0.1 M, pH 6.8)	197
							• Applied bias: −0.2 V_RHE_	
							• Light: AM 1.5G	
CuInS_2_ QDs/NiO	Charge separation and transport promoter	Immersion	CO_2_ reduction	CO	N/A	32%	• Electrolyte: Ar and CO_2_-saturated DMF	199
							• Applied bias: −1.0 V_RHE_ to −0.4 V_RHE_	
							• Light: 17 W, Zenaro Lighting GmbH, 420–750 nm	
PMAEMA/QDs	Charge transport promoter and light harvester	Electric field-driven layer-by-layer	CO_2_ reduction	HCOH	100%	N/A	• Electrolyte: CO_2_-saturated NaClO_4_ (0.1 M)	200
							• Applied bias: −0.45 V	
							• Light: 500 W Xe–Hg lamp	

#### 3.1.3. Single-atom metals

**Fig. 9:**
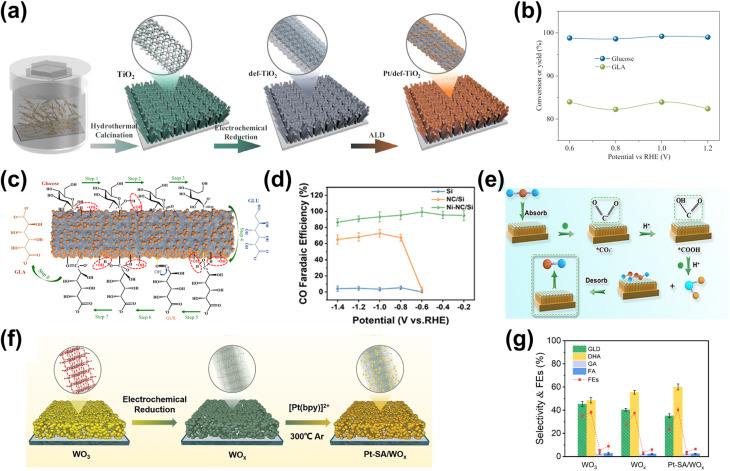
SA utilization for PEC biomass valorization selectivity enhancement and tuning. (a) Fabrication steps for Pt SAs/def-TiO_2_ NRAs. (b) Glucose conversion and selectivity of glucaric acid (GLA) as a function of applied potential. (c) Glucose oxidation mechanisms on Pt SA-modified def-TiO_2_ under light illumination and applied bias. Adapted from ref. 212. Copyright 2023 The Author(s). (d) FE of CO production on the Si-NC/Si NW photocathode. (e) Schematic of the PEC CO_2_ reduction mechanism on the Si-NC/Si NW photocathode. Adapted from ref. 213 with permission. Copyright 2024 The Royal Society of Chemistry. (f) Synthesis process of Pt–SAs/WO_*x*_. (g) Selectivity and FE of PEC glycerol oxidation on Pt–SAs/WO_*x*_ compared to WO_3_ and WO_*x*_ photoelectrodes. Adapted from ref. 214 with permission. Copyright 2024 Wiley-VCH GmbH.

The incorporation of single-atom metals (SAs) onto catalyst surfaces is a cutting-edge strategy for improving both the photoactivity and selectivity of PEC in biomass valorization. Unlike the previous two heterostructuring strategies discussed above, SAs serve as isolated, highly efficient catalytic centers due to their unique electronic and geometric characteristics.^39,204,205^ From a photoactivity perspective, SAs profoundly influence the electronic structure of the underlying catalyst.^206^ By modulating the bandgap of semiconductors or altering the electronic properties of carbon-based materials, SAs improve light absorption and optimize energy band alignment.^207–209^ This modification enhances photogenerated charge carrier separation while minimizing recombination losses, ensuring that more electrons and holes participate in catalytic reactions. Additionally, SAs can act as electron sinks or hole traps, facilitating charge carrier transfer to reactants and accelerating reaction kinetics.^210,211^ For instance, Pt SAs anchored on O_Vac_-rich TiO_2_ nanorods (Pt/def-TiO_2_ NRAs) have demonstrated exceptional selectivity in glucose oxidation to glucaric acid.^212^ Here, TiO_2_ underwent electrochemical reduction to introduce O_Vac_, which then serves as the anchoring site for Pt SAs deposited by atomic layer deposition (Fig. 9a). Furthermore, O_Vac_ in def-TiO_2_ enhances charge carrier extraction, as its large electron conduction region and substantial surface band bending promote efficient electron transport. Specifically, extracted electrons migrate to the counter electrode for H_2_ production, while the valence band structure of defective TiO_2_ optimizes hole energy for glucose oxidation. Following that, Pt SAs facilitate hole transfer to glucose, enabling selective oxidation to glucaric acid. Using this approach, a remarkable glucose conversion is achieved, *i.e.*, 98.8% conversion, with 84.3% selectivity toward glucaric acid—over six times higher than that of bare def-TiO_2_ NRAs, which predominantly yield gluconic acid (Fig. 9b). This significant shift in selectivity is attributed to the unique reaction kinetics introduced by Pt SAs. Mechanistically, it was revealed that glucose is initially oxidized to gluconic acid, which then converts into l-guluronic acid before forming glucaric acid (Fig. 9c). On bare def-TiO_2_, the charge transfer rate for gluconic acid is 45 s^−1^, whereas for l-guluronic acid, it is significantly higher at 190 s^−1^, indicating that the conversion of gluconic acid to l-guluronic acid is the rate-limiting step. The incorporation of Pt SAs reduces the charge transfer rate of gluconic acid to 90 s^−1^, thereby shifting selectivity toward glucaric acid.

Beyond improving charge transfer, SAs' unsaturated coordination environments lead to stronger interactions with reactants and intermediates. These unique active sites enable precise tuning of reaction pathways, significantly improving PEC reaction selectivity.^20^ Furthermore, SAs prevent active site aggregation, ensuring uniform activity and precise control over the catalytic process. One example of the effort to harness these properties is by dispersing Ni SAs on silicon nanowires (Si NWs), which allows the photocathode to achieve nearly 100% FE for CO_2_ reduction to CO at −0.6 V_RHE_ (Fig. 9d).^213^ This high selectivity stems from the unique electronic structure of Ni SAs. Among transition metals, Ni exhibits the lowest thermodynamic barrier for *COOH formation and CO desorption, favoring CO_2_ reduction to CO (Fig. 9e). Additionally, the high surface area of Ni SAs dispersed on Si NWs provides abundant reaction sites, further enhancing catalytic performance. In detail, under illumination, electrons in Si are excited from the corresponding VB to CB. When an external bias is applied, electrons from the CB of Si are driven toward Ni SAs, where CO_2_ reduction to CO occurs. In addition, Pt SAs can be dispersed on a WO_3_ amorphous/crystalline homojunction to selectively oxidize glycerol to DHA.^214^ The photoanode is fabricated through the electrochemical reduction of WO_3_, followed by immersion in a solution containing 2,2′-bipyridine and Pt cations ([Pt(bpy)]^2+^) and annealing (Fig. 9f). Due to its unique gradient structure, the photoanode exhibits an upward shift in *E*_F_ from the inner to the outer layer. This shift generates an internal electric field that, upon illumination, drives photogenerated charge carriers toward the photoanode/electrolyte interface, where they participate in the oxidation reaction. Additionally, this internal electric field effectively suppresses charge recombination, further enhancing the reaction efficiency. In this approach, adsorption plays a crucial role. The middle hydroxyl groups of glycerol spontaneously adsorb onto the W atom sites of the photoelectrode, while Pt SAs provide additional adsorption sites for the oxygen atoms of both the terminal and middle hydroxyl groups of glycerol. Once adsorbed, glycerol undergoes dehydrogenation, forming carbon-centered radicals as intermediates through reactions with photogenerated holes from the photoanode. The unique structure of the Pt-SA/WO_*x*_ surface significantly influences selectivity. The free energy barrier for radical formation at the middle carbon is lower than that at the terminal carbon, favoring the selective conversion of glycerol to DHA. As a result, the Pt-SA/WO_*x*_ photoanode achieves an impressive glycerol-to-DHA selectivity of up to 60.2% (Fig. 9g). Additionally, Co SAs can be integrated with C_3_N_4_/α-Fe_2_O_3_ to enhance PEC-driven syngas production from CO_2_ reduction.^215^ Although the actual syngas production occurs at a bimetallic Ag/Pd cathode, where CO_2_ chemisorption and CO desorption take place, Co SAs play a crucial role by providing active sites for C_3_N_4_ anchoring. These sites facilitate water oxidation and proton/electron transfer, supporting overall reaction efficiency.

While the application of SAs has been extensively explored and widely used in electrochemical systems,^216–219^ these examples highlight their versatility in PEC biomass valorization. Unlike nanoparticles or quantum dots, SAs serve as isolated, highly efficient catalytic centers due to their unique electronic and geometric characteristics. From a photoactivity perspective, SAs profoundly influence the electronic structure of the underlying catalyst. This modification enhances light absorption and optimizes energy band alignment, improving photogenerated charge carrier separation while minimizing recombination losses. SAs can also act as electron sinks or hole traps, facilitating charge carrier transfer to reactants and accelerating reaction kinetics. Beyond improving charge transfer, SAs' unsaturated coordination environments lead to stronger interactions with reactants and intermediates. These unique active sites enable precise tuning of reaction pathways, significantly improving PEC reaction selectivity. Furthermore, SAs prevent active site aggregation, ensuring uniform activity and precise control over the catalytic process. Their high surface area also provides abundant reaction sites. However, functionalization with SAs often comes with a high cost due to their sophisticated method.^220^ A summary of SA-based PEC biomass valorization strategies is presented in Table 4.

**Table 4:** Summary of single atom metal (SA) utilization on the surface of photoelectrodes for PEC biomass valorization selectivity enhancement and tuning applications

Photoelectrode^a^	SA role	Deposition method	Reaction	Target product(s)	Selectivity	FE	Reaction conditions	Ref.
Pt SAs/TiO_2_ NRAs	Charge transport promoter	Atomic layer deposition	Glucose oxidation	Glucaric acid	84.3%	N/A	• Electrolyte: KOH (1 M) with glucose	212
							• Applied bias: 0.6 V_RHE_	
							• Light: AM 1.5G (100 mW cm^−2^)	
Ni-NC/Si NWs	Charge transport, promoting COOH* formation and CO desorption	Drop-casting	CO_2_ reduction	CO	N/A	100%	• Electrolyte: CO_2_-saturated KHCO_3_ (0.1 M)	213
							• Applied bias: −0.6 V_RHE_	
							• Light: AM 1.5G (100 mW cm^−2^)	
Pt SAs/WO_*x*_	Charge separation and transport promoter, adsorption site for O atoms of the terminal and middle hydroxyl	Immersion	Glycerol oxidation	DHA	60.2%	N/A	• Electrolyte: Na_2_SO_4_ (0.5 M) with glycerol (0.1 M) with pH 2, adjusted by H_2_SO_4_	214
							• Applied bias: 1.2 V_RHE_	
							• Light: AM 1.5G (100 mW cm^−2^)	

a NRA: nanorod array, NW: nanowire, and SA: single atom.

#### 3.1.4. Layered double hydroxides

Layered double hydroxides (LDHs) have gained significant attention in PEC applications due to their well-defined two-dimensional layered structure, tunable internal architecture, cost-effective and non-toxic metal precursors, and adjustable composition.^221,222^ Structurally, LDHs resemble hydrotalcite, comprising positively charged brucite-like layers neutralized by intercalated anionic species and water molecules. The metal cations within LDH layers adopt an octahedral coordination, forming metal hydroxide sheets. The structural integrity of LDHs is primarily maintained by two interactions: first, coulombic attraction between the positively charged metal hydroxide layers and the interlayer anions, which depends on charge distribution, and second, hydrogen bonding between the oxygen atoms of intercalated anions and the hydroxyl groups in the metal hydroxide layers, influenced by coordination symmetry.^40,223,224^ A key advantage of LDHs is the uniform distribution of divalent (M^2+^), trivalent (M^3+^), or tetravalent (M^4+^) cations across the hydroxide layers, preventing the formation of localized aggregation sites.

**Fig. 10:**
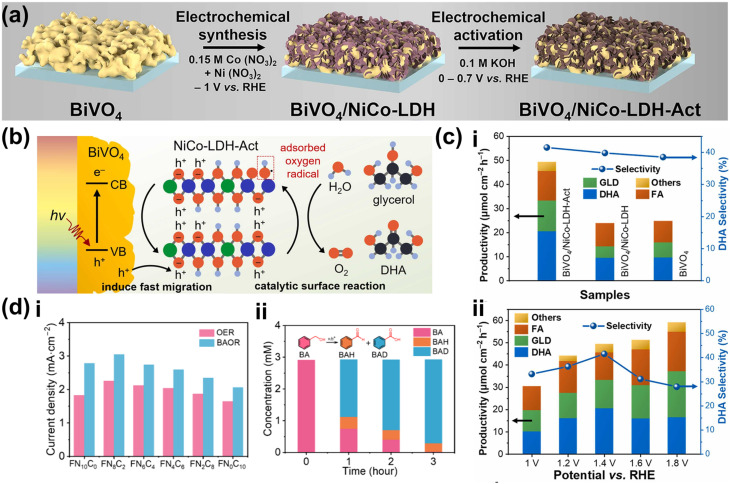
LDH–reactant interaction modulation for PEC biomass valorization selectivity control. (a) Fabrication steps for the BiVO_4_/NiCo-LDH-Act photoanode. The LDH is electrodeposited with a three-electrode configuration at −1.0 V_Ag/AgCl_ potential with 7.5 × 10^−3^ C loading. LDH activation is conducted through cyclic voltammetry from 0 to 0.7 V for 3 cycles. (b) Mechanism of glycerol adsorption and oxidation to DHA on BiVO_4_/NiCo-LDH-Act. Upon illumination, photogenerated holes facilitate the formation of OH radicals on the surface. These adsorbed OH radicals extract H atoms of glycerol, thereby promoting selective glycerol conversion to DHA. (c) Productivity of various glycerol oxidation products and DHA selectivity over BiVO_4_/NiCo-LDH-Act (i) compared to inactivated BiVO_4_/NiCo-LDH and bare BiVO_4_ photoanodes and (ii) at different potentials within 1 h. Adapted from ref. 225 with permission. Copyright 2022 Elsevier B.V. (d) Performance of the α-Fe_2_O_3_/NiCo-LDH photoanode represented as (i) current density for different Ni and Co compositions and (ii) product selectivity over 3 h of reaction. Adapted from ref. 226 with permission. Copyright 2024 American Chemical Society.

One effective approach to improving the selectivity of PEC biomass valorization is to leverage the tunable interaction of LDHs with reactants. For instance, NiCo LDHs can be employed to improve the selectivity of PEC glycerol^225^ and benzyl alcohol^226^ oxidation reactions. For the former, controlling selectivity to a higher-valued chemical remains a challenge due to frequent C–C bond cleavage and hydroxyl group peroxidation during electrooxidation. Therefore, to address this, activated NiCo-LDH can be integrated with BiVO_4_ (Fig. 10a) to produce DHA. The surface oxygen activation process significantly increases the lattice oxygen content, primarily due to the dehydrogenation of hydroxyl groups, which then exposes active oxygen sites on the LDH surface. As a result, it enhances both hole transfer efficiency and surface oxidation activity (Fig. 10b), contributing to improved selectivity to nearly 42% and a productivity of 20.5 μmol cm^−2^ h^−1^ at 1.4 V_RHE_ under light illumination (Fig. 10c(i)), which is higher than those of bare BiVO_4_ and inactivated LDH-modified photoelectrodes. Notably, when the applied bias was increased, DHA selectivity decreased, likely due to excessive glycerol oxidation, which led to C–C bond breakage (Fig. 10c(ii)). It thus demonstrates tunable selectivity for PEC glycerol oxidation. Moreover, for the selective PEC oxidation of benzyl alcohol, an α-Fe_2_O_3_ photoanode can be loaded with a NiCo-LDH cocatalyst *via* a hydrothermal method. In this approach, the performance depends on the relative Ni and Co content in NiCo-LDH (Fig. 10d). As the Co content increases, the electron density of Ni decreases, making it more susceptible to oxidation. At the same time, the energy barrier for forming high-valence species is lowered, thereby enhancing the driving force for NiCo-LDH oxidation by photogenerated holes on α-Fe_2_O_3_. This effect is crucial because if the oxidative capability of high-valence NiCo-LDH species is too weak, it becomes challenging to extract protons and electrons from alcohol molecules. As shown in Fig. 10e, the photoanode achieved a benzyl alcohol conversion efficiency of 99.1% and a benzoic acid selectivity of 90.9%. This mechanism confirms that the NiCo-LDH cocatalyst facilitates PEC benzyl alcohol oxidation through an indirect catalytic process involving high-valence species.

**Fig. 11:**
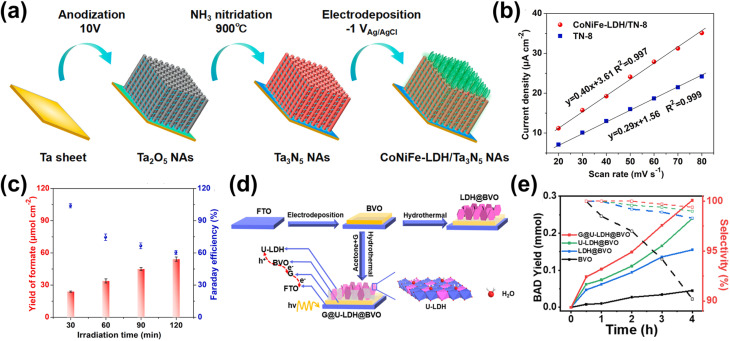
LDH-improved active surface area to enhance PEC biomass valorization selectivity. (a) Fabrication steps of the CoNiFe-LDH/Ta_3_N_5_ photoanode. (b) Plots of current density as a function of scan rate for the bare Ta_3_N_5_ (blue) and CoNiFe-LDH/Ta_3_N_5_ photoelectrodes. The electrochemically active surface area (ECSA) is estimated by double-layer capacitance, which is represented by a linear slope. The ECSA of LDH-modified Ta_3_N_5_ is calculated to be 1.38 times that of bare Ta_3_N_5_. (c) Yield of formate and the corresponding FE for formate on CoNiFe-LDH/Ta_3_N_5_. Adapted from ref. 227 with permission. Copyright 2021 Elsevier Ltd. (d) Fabrication process of graphene@ultrathin-CoAl-LDH@BiVO_4_. BiVO_4_ particles were synthesized on fluorine-doped tin oxide substrates using an electrodeposited-Bi precursor method. Ultrathin LDH nanosheets were vertically grown on the BVO surface *via* a conventional hydrothermal method, forming the ultrathin-CoAl-LDH@BiVO_4_ photoanode. To further enhance charge transfer, acetone and graphene dispersion were introduced during the hydrothermal process, resulting in the formation of the graphene@ultrathin-CoAl-LDH@BiVO_4_ photoanode. (e) Benzaldehyde yield and selectivity as a function of reaction time under illumination at an applied potential of 1.2 V_RHE_. Adapted from ref. 228 with permission. Copyright 2020 American Chemical Society.

Aside from modifying LDH interaction with reactants, LDHs can lead to an increased active surface area, which will improve the PEC performance of the system. For instance, trimetallic transition metal CoNiFe-LDH can be electrodeposited onto Ta_3_N_5_ nanotube arrays (Fig. 11a).^227^ Through this approach, the trimetallic CoNiFe-LDH nanosheets (NSs) exhibit an enhanced specific surface area, a higher density of exposed active sites, and reduced charge-transfer resistance, attributed to their ultrathin 2D nanostructure and optimized electronic properties (Fig. 11b). This strategy successfully enhances the selectivity of the PEC GOR, achieving nearly 100% FE for the co-production of formate and H_2_ (Fig. 11c). The improved selectivity stems from the efficient transfer of photogenerated holes in Ta_3_N_5_ to the photoelectrode/electrolyte interface, which is assisted by CoNiFe-LDH. Furthermore, the surface-adsorbed glycerol molecules are then activated by these holes and converted into formic acid through further oxidation, which involves C–C bond cleavage. The unique structure of CoNiFe-LDHs and their ability to increase active sites result in improved adsorption and interaction between glycerol and the photoanode. Combining the strategies mentioned above, an ultrathin Co-based layered double hydroxide catalyst and graphene can be incorporated into BiVO_4_ photoanodes using a hydrothermal method followed by acetone treatment to selectively oxidize benzyl alcohol to benzaldehyde (Fig. 11d).^228^ The additional acetone treatment promotes the specific growth needed to form ultrathin LDH nanosheets. With this approach, a benzaldehyde yield of 289 μmol was achieved in 4 hours, with a selectivity of nearly 100% (Fig. 11e). This impressive performance stems from the capabilities of the LDH to lower their adsorption energy by adsorbing OH radicals through hydrogen bonding. Furthermore, under illumination, the benzyl alcohol adsorbs onto the LDH surface and converts to carbon-centered radicals, which then combine with the relayed OH radicals obtained by the oxidation of water, and the C–O bond was activated to produce benzaldehyde.

**Fig. 12:**
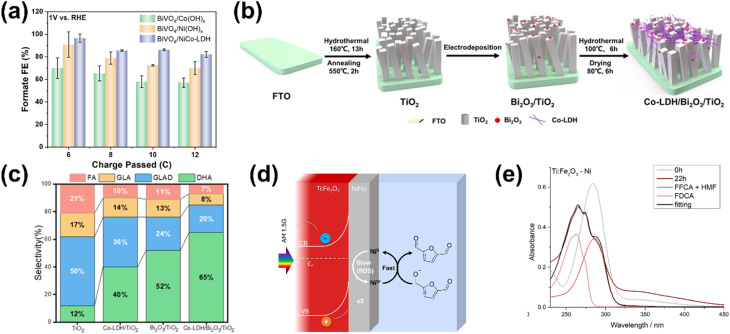
Utilization of LDHs and hydroxides as charge transfer promoters to improve PEC biomass valorization selectivity. (a) FE of formate from ethylene glycol oxidation on the BiVO_4_/NiCo-LDH, BiVO_4_/Ni(OH)_*x*_ and BiVO_4_/Co(OH)_*x*_ photoanodes at an applied bias of 1 V_RHE_. Adapted from ref. 229 with permission. Copyright 2024 Wiley-VCH GmbH. (b) Fabrication process of the Co-LDH/Bi_2_O_3_/TiO_2_ photoanode. (c) Selectivity of glycerol oxidation products of Co-LDH/Bi_2_O_3_/TiO_2_ compared to other photoanodes. Adapted from ref. 230 with permission. Copyright 2025 American Chemical Society. (d) Ni(OH)_2_-deposited Ti:Fe_2_O_3_ for selective HMF oxidation to FDCA. (e) UV-vis absorbance spectra evolution from 0 to 22 h of HMF reaction on Ni(OH)_2_/Ti:Fe_2_O_3_.

Another role LDHs play in improving the selectivity of PEC biomass valorization is enhancing charge transfer. For instance, BiVO_4_/NiCo-LDH is shown to improve the selectivity for PEC ethylene glycol oxidation, a key process for converting hydrolysis byproducts of polyethylene terephthalate (PET) plastic waste. Specifically, the heterostructure achieves an impressive FE of over 90% for formate in 0.1 M KOH (Fig. 12a),^229^ attributed to the synergistic effect of Ni(OH)_*x*_ and Co(OH)_*x*_. On one hand, Ni(OH)_*x*_ provides a high number of active sites for ethylene glycol oxidation. On the other hand, Co(OH)_*x*_ on BiVO_4_ acted as a protective layer. Furthermore, when combined, the generation and transfer of photoexcited holes from BiVO_4_ to NiCo-LDH lead to the oxidation of Ni^2+^ and Co^2+^ to higher oxidation states (Ni^3+^ and Co^3+^ or beyond), further catalyzing ethylene glycol oxidation. In addition, Co-LDH, alone, can be utilized to improve the selectivity of glycerol oxidation toward high-value products, particularly DHA.^230^ This approach involves developing Bi_2_O_3_ on TiO_2_ nanorod arrays *via* electrodeposition, followed by the hydrothermal synthesis of linear Co-LDH on Bi_2_O_3_/TiO_2_ (Fig. 12b). Through this method, a dual type-II heterojunction is formed, effectively suppressing electron–hole recombination. The resulting photoanode allows PEC glycerol oxidation to achieve 65% selectivity, surpassing the 52% for bare Bi_2_O_3_/TiO_2_ (Fig. 12c).

Beyond layered double hydroxides, the use of hydroxides such as Ni(OH)_2_ offers a promising strategy to tune the selectivity of HMF oxidation. In one such system, Ni(OH)_2_ is deposited on the surface of titanium-doped hematite (Ti:Fe_2_O_3_) (Fig. 12d).^231^ Fe_2_O_3_ was chosen for its inherent stability, cost-effectiveness, and excellent light-harvesting capabilities in the visible spectrum.^232^ However, its widespread application is often hampered by limited hole diffusion and charge injection efficiency. The introduction of the Ti dopant effectively addresses these limitations by improving charge carrier transport and significantly reducing the recombination of photogenerated charge carriers.^233^ Furthermore, the deposition of Ni(OH)_2_ leads to the swift utilization of excess holes on the oxidized nickel sites by HMF, as evidenced by the nickel oxidation state as a function of applied potential. This strong hole utilization is crucial because it effectively diverts photogenerated holes towards HMF oxidation, thereby suppressing the competitive OER and directly enhancing the selectivity toward HMF oxidation. This directly enhances the selectivity towards HMF oxidation over water oxidation. From a reaction kinetics perspective, the regeneration of Ni^4+^ from its reduced state is the rate-determining step. The decrease in the nickel oxidation state upon HMF addition indicates that Ni^4+^ is rapidly reduced through interaction with HMF. Through the evolution of the absorption spectra (Fig. 12e), it was calculated that the deposition of Ni(OH)_2_ results in up to 49% selectivity towards FDCA.

To summarize, these examples highlight the versatility of LDHs as cocatalysts in selective PEC biomass valorization reactions. With the ability to modify the binding energy of molecule species and enhance charge transport, LDHs offer a promising solution to improve the selectivity of PEC biomass valorization. Despite their promising advantages, LDHs also present several limitations that can hinder their practical application in PEC biomass valorization. One key drawback is the precise control over the composition and crystallinity of LDHs, particularly when scaling up synthesis for practical applications. These limitations underscore the need for continued optimization of LDH synthesis, stabilization, and integration strategies in PEC systems. The utilization of LDHs in PEC biomass valorization is summarized in Table 5.

**Table 5:** Summary of layered double hydroxide (LDH) utilization on the surface of photoelectrodes for PEC biomass valorization selectivity enhancement and tuning applications

Photoelectrode^a^	LDH role	Deposition method	Reaction	Target product(s)	Selectivity	FE	Reaction conditions	Ref.
BiVO_4_/NiCo-LDH-Act	Promoting surface oxidation and charge transfer promoter	Electrodeposition and CV-assisted activation	Glycerol oxidation	DHA	42%	N/A	• Electrolyte: Na_2_SO_4_ (0.5 M) with glycerol (0.6 M)	225
							• Applied bias: 1.4 V_RHE_	
							• Light: AM 1.5G (300 W Xe lamp)	
α-Fe_2_O_3_/NiCo-LDH	Promoting high-valence species formation and surface oxidation	Hydrothermal	Benzyl alcohol oxidation	Benzoic acid	90.9%	N/A	• Electrolyte: KOH (1 M) with benzyl alcohol (50 mM)	226
							• Applied bias: 1.23 V_RHE_	
							• Light: AM 1.5G (300 W Xe lamp)	
CoNiFe-LDHs/Ta_3_N_5_ NAs	Promoting glycerol adsorption and C–C bond breaking, charge transfer promoter	Electrodeposition	Glycerol oxidation	Formate	98%	N/A	• Electrolyte: NaOH (0.1 M)	227
							• Applied bias: 1.4 V_RHE_	
							• Light: AM 1.5G (Xe lamp)	
Graphene@ ultrathin-CoAl-LDH @BiVO_4_	Promoting benzyl alcohol and OH radical adsorption, charge transfer promoter	Hydrothermal	Benzyl alcohol oxidation	BAD	99%	N/A	• Electrolyte: PBS (0.1 M, pH 7) with benzyl alcohol (2 mM)	228
							• Applied bias: 1.2 V_RHE_	
							• Light: AM 1.5G (100 mW cm^−2^)	
BiVO_4_/NiCo-LDH	Charge transfer promoter	Hydrothermal	Ethylene glycol oxidation	Formate	N/A	85%	• Electrolyte: KOH (0.1 M)	229
							• Applied bias: 1.0 V_RHE_	
							• Light: AM 1.5G	
Co-LDH/Bi_2_O_3_/TiO_2_	Charge transfer promoter	Hydrothermal	Glycerol oxidation	DHA	65%	N/A	• Electrolyte: Na_2_SO_4_ (0.1 M) with glycerol (0.1 M)	230
							• Applied bias: 1.23 V_RHE_	
							• Light: AM 1.5G	

a Act: activated and NA: nanotube array.

#### 3.1.5. Molecular co-catalysts

The integration of molecular co-catalysts, such as coordination compounds, into PEC systems offers a sophisticated strategy for enhancing photoconversion efficiency and selectivity in biomass valorization. These molecular complexes, typically comprising transition metals coordinated with ligands, function as highly specialized catalytic centers that facilitate targeted redox reactions. Their molecular nature allows for precise tuning of redox potential, coordination environment, and electronic structure to align with the specific requirements of PEC reactions.^175–178^ While most current applications of such systems have focused on PEC water splitting, their potential for biomass valorization is equally promising. In particular, immobilizing or attaching molecular co-catalysts onto the surface of photoelectrodes enhances photoconversion efficiency by mediating charge transfer between the photoelectrode and the reactants. Acting as intermediaries, these surface-bound co-catalysts accept photogenerated electrons or holes from the photoelectrode and transfer them with high precision to the adsorbed biomass molecules. This process reduces surface charge recombination, thereby ensuring that a greater proportion of photogenerated carriers contribute effectively to the desired reactions.^179–182^

**Fig. 13:**
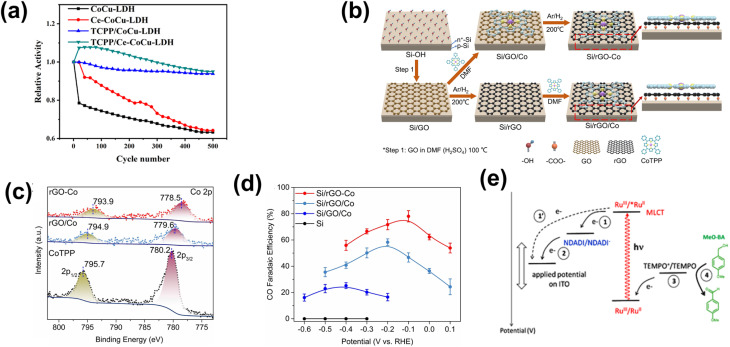
Molecular co-catalyst utilization to improve catalytic activity for enhanced PEC biomass valorization selectivity. (a) Reactivity stability of bare and modified CoCu-LDHs over 500 cycles for methanol oxidation. Adapted from ref. 234 with permission. Copyright 2022 American Chemical Society. (b) Preparation steps for Si/GO/Co, Si/rGO-Co and Si/rGO/Co photocathodes. (c) XPS spectra of Co 2p for Si/GO/Co, Si/rGO-Co and Si/rGO/Co photocathodes. The peak shift is bigger for Si/rGO-Co compared to Si/rGO/Co, indicating that the electronic interaction of the former is stronger than that of the latter, which aligns with its improvement in selectivity towards CO from PEC CO_2_ reduction. (d) FE of CO from CO_2_ reduction over various photocathodes as a function of applied bias. Adapted from ref. 235 with permission. Copyright 2023 Wiley-VCH GmbH. (e) Ru-based molecular co-catalyst structure used to modify the α-Fe_2_O_3_ photoanode surface. Ru_2_ and Ru_4_ are synthesized as the carboxylic derivatives of Ru_1_ and Ru_3_, respectively, to enable the anchoring process on the surface of α-Fe_2_O_3_. The immobilization is conducted by stirring α-Fe_2_O_3_ with methanol solution containing the molecular co-catalysts.

As an example, anchoring 5,10,15,20-tetrakis(4-carboxylphenyl)porphyrin (TCPP) molecules onto Ce–CoCu-LDH has been shown to enhance PEC methanol oxidation.^234^ Here, TCPP exhibits high catalytic activity and durability, consequently increasing the light absorption capability of the photoelectrode and generating more photogenerated electrons. Moreover, rapid charge transfer from TCPP to Ce–CoCu-LDH mitigates charge recombination, leading to an enhancement in reactivity by up to 2.3 times even after 500 cycles (Fig. 13a). The method of immobilizing molecular catalysts onto the photoelectrode also plays a crucial role in optimizing charge transfer. For example, modifying the preparation steps allows Si/rGO photoelectrodes to be loaded with a Co-based molecular co-catalyst in different stacking configurations, yielding varying performances.^235^ In one strategy, cobalt tetraphenylporphyrin (CoTPP) is immobilized on Si/GO and Si/rGO photoelectrodes through impregnation adsorption in DMF, followed by either separate or simultaneous graphene reduction and calcination (Fig. 13b). This approach enables the photoelectrode to achieve an impressive 82.8% FE for CO from PEC CO_2_ reduction at a near-neutral potential of −0.1 V_RHE_. The simultaneous reduction and calcination process strengthens the electronic interaction between rGO and CoTPP, as evidenced by the shifted Co binding signals (Fig. 13c). Consequently, enhanced electronic interaction improves electron transfer kinetics and catalytic activity, translating to a superior CO selectivity of up to 82.8% in CO_2_ reduction at −0.1 V_RHE_ (Fig. 13d). In addition, the catalytic activity of molecular cocatalyst-modified photoelectrodes can be improved by creating synergistic interfaces.^236^ The formation of these synergistic interfaces is possible due to the modularity of coordination compounds of the molecular cocatalysts. One notable example is the improved oxidation of *para*-methoxy benzyl alcohol (MeO-BA) to *para*-methoxy benzaldehyde using a catalyst-dye-acceptor molecular array.^237^ This array comprises a Ru tris–bipyridine complex as a photosensitizer, linked at one end to 2,2,6,6-tetramethyl-1-piperidine *N*-oxyl (TEMPO) as an alcohol oxidation catalyst, and at another end to the electron acceptor naphthalene-dicarboxyanhydride-dicarboximide (NDADI). Upon illumination, the Ru complex undergoes metal-to-ligand charge transfer, followed by electron injections either directly to the substrate (ITO NPs) or to NDADI. In the former case, the hole shifts from Ru^III^ to TEMPO, driving the oxidation reaction. In the latter case, an additional electron transfer occurs before the hole transfer to TEMPO and subsequent catalysis (Fig. 13e). This synergistic architecture enables the photoelectrode to achieve an outstanding 80% FE for *para*-methoxy benzaldehyde, a significant improvement from the initial 28% FE without TEMPO. This selectivity enhancement results from the combination of the highly oxidative Ru complex and the efficient hole transfer to TEMPO.

**Fig. 14:**
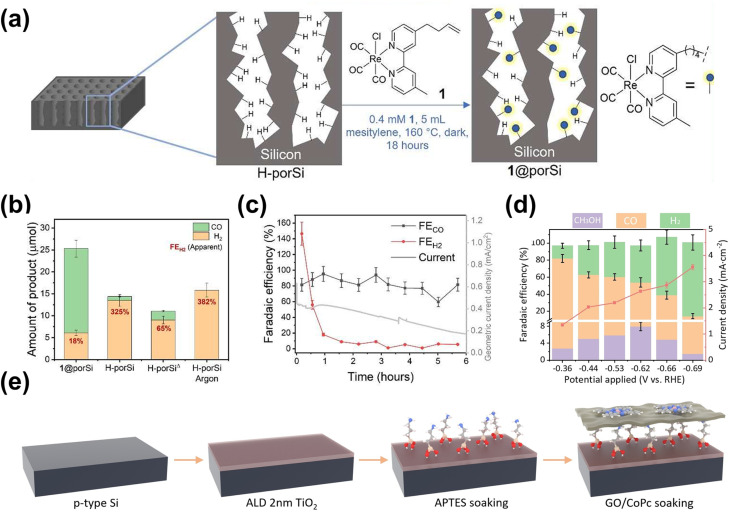
Molecular co-catalyst-enabled PEC biomass valorization selectivity tuning. (a) Immobilization step of 1 onto porSi to form a 1@porSi photoelectrode. (b) Comparison of product yields and FE on 1@porSi compared to other photoelectrodes. (c) Performance of 1@porSi represented as the evolution of FE over 6 h of PEC CO_2_ reduction reaction. Adapted from ref. 241 with permission. Copyright 2024 American Chemical Society. (d) Fabrication process of STA-GO/CoPc. (e) PEC CO_2_ reduction of STA-GO/CoPc performance represented as FE over varying applied potentials. Adapted from ref. 243 with permission. Copyright 2022 Wiley-VCH GmbH.

In the domain of selectivity tuning, molecular co-catalysts provide well-defined active sites with tunable electronic and geometric properties, enabling selective interactions with specific reactants or intermediates.^41,238,239^ This targeted interaction minimizes the formation of undesired by-products and enhances the yield of high-value products. One effective strategy to exploit these capabilities is the integration of a photoelectrode with a molecular co-catalyst tailored for specific reactions. For instance, (bpy)Re(CO)_3_Cl is commonly employed to enhance CO_2_ reduction selectivity toward CO by suppressing formate formation while promoting CO production.^240^ This selectivity arises from the unique reaction pathway in which formate is generated through a hydride reaction at an anion coordination site, whereas CO_2_ fixation occurs at another site of the Re complex, thereby preventing formate production due to coordinating anions. A practical approach to leveraging this strategy involves coupling porous p-Si with (bpy)Re(CO)_3_Cl *via* hydrosilylation (*i.e.*, the addition of Si–H across unsaturated C=C or C=O bonds), forming a (bpy)Re(CO)_3_Cl@porSi (1@porSi) photoelectrode (Fig. 14a).^241^ Planar p-Si is electrochemically etched using a hydrofluoric acid solution in ethanol with a 40 mA current to create a porous morphology. This increases the active surface area, enabling greater co-catalyst loading and thereby improving the reactivity and selectivity of CO_2_ reduction. Under AM 1.5G illumination at −1.71 V_Fc^+^/Fc_ in CO_2_-saturated acetonitrile containing phenol, the photoelectrode achieved an average FE of 90% for CO, significantly outperforming pristine porous p-Si and exhibiting a five-fold improvement over Re complex-deposited planar p-Si (Fig. 14b), with high durability (Fig. 14c). Notably, the 1@porSi photoelectrode maintained a FE above 70% even after 5.5 hours of reaction, whereas the catalyst-deposited planar p-Si exhibited activity for only 15 minutes. This high selectivity can be attributed to the substantial molecular catalyst loading, with 41 nmol of Re per 1.2 cm^2^ of the 1@porSi photoelectrode—approximately 100 times greater than that of planar p-Si. Moreover, the Re complex demonstrates excellent stability, with minimal leaching observed, as only 10% of the initial catalyst was found in solution after extended operation. Another promising approach involves the integration of mesoporous TiO_2_-coupled p-Si (Si/mesoTiO_2_) with phosphonated cobalt(ii) bis(terpyridine) (CotpyP) molecular catalysts *via* an immersion process to enhance PEC CO_2_ reduction.^242^ The mesoporous TiO_2_ interlayer facilitates high CotpyP loading, improving electron transfer efficiency. Under sunlight irradiation, photogenerated carriers are transferred from Si to the meso-TiO_2_ layer and subsequently to the adsorbed molecular catalysts. The catalytic process involves the formation of [Co^I^]^*n*−1^ molecular species through two possible pathways, depending on electrolyte water content: direct proton-coupled electron transfer or an indirect chemical step *via* [Co^I^]^*n*−2^ intermediates. Following CO_2_ adsorption and electron transfer, [Co^I^]^*n*−2^ transforms into [Co^II^–CO_2_^−^]^*n*−2^, a key CO-releasing intermediate. Additionally, de-coordination of pyridines stabilizes [Co^II^–CO_2_^−^]^*n*−2^*via* protonation of nitrogen atoms from deprotonated phosphonic acid, which serves as a proton relay to facilitate selective CO_2_ reduction to CO rather than formate. This system achieved an FE of up to 48% for CO under AM 1.5G irradiation at −1.0 V_Fc^+^/Fc_ in anhydrous acetonitrile containing 40% water.

Molecular co-catalysts also enable multi-electron transfer reactions, a challenge in conventional PEC systems.^244^ Their ability to sequentially store and transfer multiple electrons or holes allows them to drive complex transformations, such as the reduction of oxygenated biomass intermediates or selective oxidation processes.^245,246^ A notable example is the utilization of cobalt phthalocyanine (CoPc) as a molecular co-catalyst on Si–TiO_2_-APTES substrate-supported graphene oxide (STA-GO) for selective PEC CO_2_ reduction to CO and methanol (Fig. 14e).^243^ This architecture achieves faradaic efficiencies of 86% and 8% for CO and methanol, respectively (Fig. 14f). The CoPc catalyst facilitates the six-electron reduction of CO_2_, enabling methanol formation at higher applied bias. However, the relatively low methanol faradaic efficiency is not due to CoPc's catalytic limitations but rather to weak CO trapping on the Si surface, which favors CO production. Thus, modifying the planar Si structure to a more CO-adsorptive configuration could further enhance methanol selectivity.

To summarize, integration of molecular co-catalysts into PEC systems offers a highly effective strategy for enhancing and tuning the selectivity of biomass and CO_2_ valorization reactions. These co-catalysts provide several key advantages, such as precise tunability of redox properties and coordination environments to match specific reaction requirements, enhanced charge transfer kinetics that suppress recombination and improve carrier utilization, well-defined active sites that enable high selectivity toward desired products, and support for complex multi-electron reactions that are otherwise challenging in PEC systems. Despite their considerable advantages, the practical application of molecular co-catalysts in PEC systems is not without limitations. One critical challenge is their long-term operational stability, particularly under harsh photoelectrochemical conditions, where prolonged exposure to light, potential cycling, or aqueous media may lead to photodegradation and ultimately loss of catalytic activity.^247^ Additionally, achieving robust and uniform immobilization of molecular co-catalysts on semiconductor surfaces often requires intricate synthetic protocols or surface modifications that may not be easily scalable. Furthermore, many high-performing molecular co-catalysts incorporate rare or expensive transition metals, which could limit their economic viability for large-scale deployment.^248^ Therefore, while molecular co-catalysts offer an excellent platform for advancing PEC selectivity and activity, overcoming these challenges remains essential for their transition from laboratory-scale systems to practical biomass and CO_2_ valorization technologies. Despite these limitations, molecular co-catalysts remain a promising frontier for advancing selective and efficient PEC valorization technologies. The utilization of molecular cocatalysts for PEC biomass and CO_2_ valorization selectivity enhancement and tuning is summarized in Table 6.

**Table 6:** Summary of molecular co-catalyst utilization on the surface of photoelectrodes for PEC biomass valorization selectivity enhancement and tuning applications

Photoelectrode	Molecular cocatalyst function	Deposition method	Reaction	Target product(s)	Selectivity	FE	Reaction conditions	Ref.
Si/rGO-Co	Charge transfer promoter	Calcination	CO_2_ reduction	CO	82.8%	N/A	• Electrolyte: CO_2_-saturated KHCO_3_ (0.1 M)	235
							• Applied bias: 0.1 V_RHE_	
							• Light: AM 1.5G (100 mW cm^−2^)	
NDADI-P-Ru-TEMPO	Oxidation sites and charge transfer promoter	Chemical activation, ligand reaction, and complexation	*Para*-methoxy benzyl alcohol oxidation	*Para*-methoxy	N/A	80%	• Electrolyte: CH_3_CN with Bu_4_NPF_6_ (0.1 M) with 50 mM *para*-methoxy benzyl alcohol	237
				Benzaldehyde				
							• Applied bias: 0.4 V_SCE_	
							• Light: AM 1.5G	
(bpy)Re(CO)_3_Cl@porSi	CO_2_ reduction sites and charge transfer promoter	Hydrothermal	CO_2_ reduction	CO	N/A	90%	• Electrolyte: CO_2_-saturated MeCN with 100 mM phenol	241
							• Applied bias: −1.71 V_Fc^+^/Fc_	
							• Light: AM 1.5G (100 mW cm^−2^)	
Si/mesoTiO_2_/CotpyP	CO_2_ reduction and CO desorption intermediate, charge transfer promoter	Immersion	CO_2_ reduction	CO	N/A	48%	• Electrolyte: CO_2_-saturated TBABF_4_ (0.1 M) with 40% water:MeCN mixture	242
							• Applied bias: 1.0 V_Fc^+^/Fc_	
							• Light: AM 1.5G (100 mW cm^−2^)	
STA-GO/CoPc	Facilitating six-electron reduction process	Ultrasonication	CO_2_ reduction	CO and methanol	N/A	86% (CO) and 23% (methanol)	• Electrolyte: CO_2_-saturated KHCO_3_ (0.1 M)	243
							• Applied bias: −0.28 V_RHE_ (CO) and −1.0 V_RHE_ (methanol)	
							• Light: 1.5 sun illumination	

### 3.2. Crystal facet tuning

**Fig. 15:**
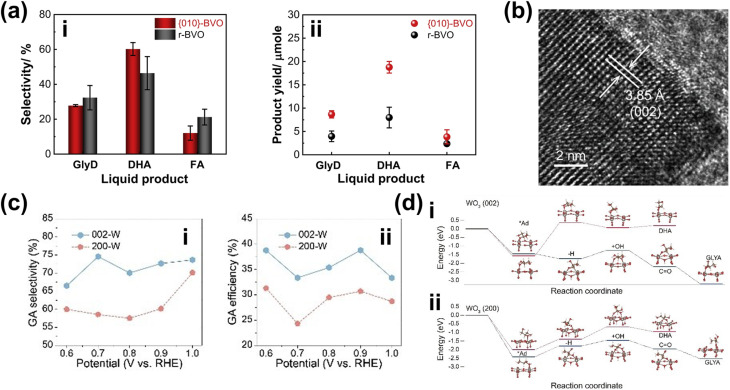
Crystal facet tuning to modulate adsorption behavior and enhance charge transport for improved PEC biomass valorization selectivity. (a) PEC glycerol oxidation performance on (010)-dominant BiVO_4_ compared to regular BiVO_4_ (r-BiVO_4_) represented by (i) product selectivity and (ii) yield. The (010) and (121) facet-dominated BiVO_4_ is fabricated through a solvent-assisted hydrothermal method. The distinction of the process is the difference in reaction pH (0.9 and 0.4 for (121) and (010)-BiVO_4_, respectively). Adapted from ref. 259 with permission. Copyright 2020 Elsevier B.V. (b) HR-TEM image of (002)-exposed WO_3_. (c) PEC glycerol oxidation (i) selectivity and (ii) FE of GA on (002)-exposed WO_3_ compared to (200)-exposed WO_3_. (d) Free energy diagram of PEC glycerol oxidation on top of (i) the (002) facet and (ii) the (200) facet of WO_3_. Adapted from ref. 260 with permission. Copyright 2023 Wiley-VCH GmbH.

Crystal facet tuning of catalytic materials in PEC of biomass has been widely explored to enable precise control over catalytic activity, selectivity, and efficiency of the reaction system.^184,249,250^ Typically, this is achieved by modulating the exposure of specific crystallographic planes on the catalyst surface. Each crystal facet possesses unique atomic arrangements, coordination states, and surface energies, which govern how reactants and intermediates interact with the catalyst.^46,251^ By strategically exposing high-energy facets, which feature more unsaturated and reactive atomic sites, the activation of biomass molecules and subsequent chemical transformations can be significantly enhanced. These facets promote stronger adsorption and stabilization of specific intermediates and are ultimately responsible for lowering activation barriers and accelerating desired reaction pathways.^252^ In contrast, lower-energy facets with more stable atomic arrangements may favor selective desorption of products. Consequently, this would reduce secondary reactions and enhance product selectivity.^253–257^ In particular, a facet with an optimized surface energy can selectively adsorb oxygen-containing intermediates from biomass feedstocks, which could stabilize reaction pathways that lead to valuable chemicals or fuels.^258^ At the same time, it can suppress the binding of competing or undesired species, minimizing side reactions and waste by-products. For example, a study highlights that exposure of different crystal facets in monoclinic BiVO_4_ was found to affect its catalytic activity in PEC of glycerol oxidation.^259^ BiVO_4_ with (010)-dominant facets exhibited superior performance to (121)-dominant BiVO_4_, achieving nearly 60% selectivity to DHA (Fig. 15a). Furthermore, the study also demonstrates that the yield of liquid products obtained from the reaction was also nearly twice as high (Fig. 15b). The superiority of the (010) facet was attributed to its ability to enable more efficient adsorption of glycerol and facilitate charge transfer ability.

In addition to altering adsorption behavior, crystal facet tuning could also significantly influence both electronic and optical properties of the photocatalyst, enhancing the overall PEC performance.^46,134,261^ Certain facets possess distinct electronic structures, such as a higher density of surface states or better band edge alignment, which facilitate efficient separation and transfer of photogenerated charge carriers. Electrons and holes may preferentially migrate along specific facets and allow photogenerated carriers to reach reaction sites with minimal recombination losses.^262^ Many studies reported that facets with favorable electronic structures may also reduce bandgaps or introduce localized surface states, which enhance the ability to absorb a broader light spectrum.^263,264^ Moreover, facets with high surface energy can amplify localized electric fields. Consequently, it could enhance the activation energy of specific reaction pathways and further improve product selectivity. For instance, monoclinic WO_3_ nanosheets with exposed [002] facets were proven to be effective for selective PEC oxidation of glycerol to GA (Fig. 15c).^260^ A photocurrent density of 1.7 mA cm^−2^ was achieved with 73% GA selectivity and 39% FE at 0.9 V_RHE_ under AM 1.5G illumination (Fig. 15d). Both experimental and theoretical analyses revealed that the superior performance of (002) facets is attributed to better charge separation, prolonged carrier lifetime from abundant surface trapping states, a lower energy barrier for the glycerol-to-GA reaction, more active sites, and stronger oxidative power of photogenerated holes. This was further confirmed by DFT calculation, which revealed that the glycerol oxidation mechanisms—for the production of both GA and DHA—are modified with the exposure of the (002) crystal facet in WO_3_. WO_3_ with exposed (002) facets exhibited a much lower energy barrier for glycerol oxidation than that with (200) facets (Fig. 15d(i and ii)). This is primarily due to the ability of the (002) surface to form a spontaneous and strong electrostatic interaction between W^6+^ and –OH with the terminal and/or middle hydroxyl groups of glycerol molecules. Consequently, this would activate glycerol to further dissociate into H^+^ and a glycerol radical, which ultimately transform into GA.

**Fig. 16:**
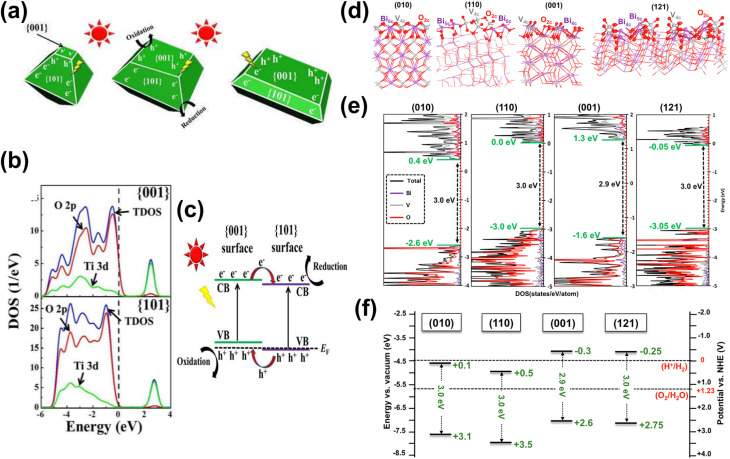
Enhancing catalytic activity through crystal facet tuning. (a) Schematic illustration of charge separation of electrons and holes in TiO_2_'s (001) and (101) facets. (b) Density of states (DOS) of TiO_2_'s (001) and (101) facets. (c) Illustration of the formation of a surface heterojunction. Adapted from ref. 266 with permission. Copyright 2014 American Chemical Society. (d) Relaxed slab structures, (e) DOS diagram, and (f) VBM/CBM positions and bandgap of monoclinic BiVO_4_ surfaces at various crystal facets. The role of each facet in proton reduction was linked to the surface Bi coordination number and its geometrical distribution. The study reveals that the (001) facet is the most suitable for both the HER and the OER, whereas the (010) facet is effective only for the OER. The (110) and (121) facets were also identified as viable for the OER, though less favorable than (001) and (010). Adapted from ref. 267. Copyright 2020 American Chemical Society.

The catalytic performance of facet-tuned anatase nanocrystals with different shapes, *i.e.* rectangular, rhombic, and nanobar, for hydrogen production *via* methanol photo-steam reforming has also been studied.^265^ Ti^3+^ defect concentration was found to strongly correlate with hydrogen evolution rates. Rectangular nanocrystals with (001), (101), and (010) facets exhibited the highest activity due to the formation of a surface heterojunction that enhances charge separation by directing electrons to (101) and (010) facets and holes to (001) facets. This study highlights the crucial role of facet-dependent electron transfer and photogenerated defects in optimizing anatase nanocrystal photocatalysis. In another study, the effect of the ratio for (001) and (101) facets in anatase TiO_2_ on its photocatalytic ability was also theoretically calculated using DFT.^266^ Here, results from density of states (DOS) calculation support the introduction of the “surface heterojunction” concept, which explains the enhanced photocatalytic activity arising from the interaction between co-exposed (001) and (101) facets (Fig. 16a–c). A similar phenomenon was also observed on BiVO_4_, where the photocatalytic activity was significantly influenced by the exposure of certain crystal facets.^267^ According to the report, it was revealed that both the band positions, valence band maximum (VBM) and conduction band minimum (CBM), and the bandgap of BiVO_4_ were found to be highly influenced by the exposure of certain crystal faces (Fig. 16d–f). Here, the exposure of (010), (110), (001), and (121) facets of monoclinic BiVO_4_ was believed to greatly influence redox and transport properties despite similar electronic structures. As a result, the (001) facet was found to be the most suitable for the HER and OER, while the (010) facet is effective primarily for the OER. These studies highlight how facet tuning, through the interplay of surface energy, adsorption energy, and electronic structure, enables precise modulation of redox reactions, improving the tunability of PEC systems.^268^ This approach therefore offers new insights into optimizing photoelectrodes for selective PEC biomass valorization.

**Fig. 17:**
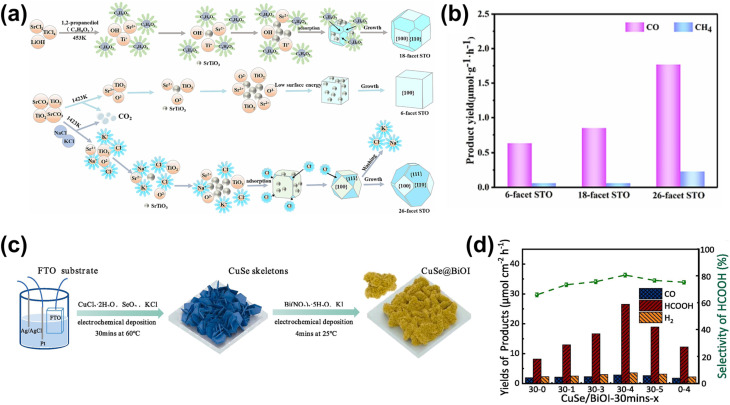
Crystal facet tuning-induced spatial charge separation and isotropic charge transport to improve PEC biomass valorization selectivity. (a) Schematic of the molten salt method for the preparation of facet-exposed STO. The formation of facet-exposed STO is driven by the selective adsorption of Cl^−^ ions on the high-energy (111) facets, reducing their surface energy and stabilizing the final structure. During molten salt treatment, STO nanoparticles grow through Ostwald ripening, with high-energy facets shrinking while low-energy facets dominate. Similarly, in the one-step molten salt method, Sr^2+^ and O^2−^ ions migrate to TiO_2_ surfaces, forming STO nuclei, with Cl^−^ adsorption guiding the exposure of (111) facets. (b) Product yield of photocatalytic CO_2_ reduction on 6-, 18-, and 26-facet exposed STO. Adapted from ref. 269 with permission. Copyright 2023 Elsevier B.V. (c) Preparation steps for the (102) facet-exposed CuSe@BiOI heterojunction. (d) PEC CO_2_ reduction product yield and selectivity of FA on CuSe@BiOI with varying deposition times. Complementing the (102)-dominant CuSe@BiOI, the porous structure of the photoelectrodes allows more CO_2_ adsorption and activation. Adapted from ref. 270 with permission. Copyright 2024 Elsevier B.V.

Furthermore, crystal facet tuning is also capable of enhancing spatial charge separation in the photoelectrode. For instance for SrTiO_3_ (STO), a total of 26 crystal facets with unique co-exposure of (100)/(110)/(111) were synthesized using the molten salt method (Fig. 17a).^269^ Based on the results from *in situ* photodeposition experiments, it was revealed that the active reaction sites for reduction and oxidation were distributed based on the anisotropic distribution of the (100) facet and (111)/(110) facets, respectively. This is primarily due to the preferential distribution of photogenerated charge carriers, where electrons were found to migrate to (100) facets and holes to (111)/(110) facets. Consequently, such facet-induced internal electric fields significantly improved carrier separation and enhanced the photocatalytic activity of the as-prepared STO for CO_2_ reduction to CO and methane (Fig. 17b). In one case, enhancement of catalytic efficiency is caused by a phenomenon called isotropic charge transport, which typically occurs when the photogenerated charge carrier moves in all directions on a specific facet.^270^ For example, a porous CuSe@BiOI heterojunction catalyst with enriched (102) facets was successfully synthesized *via in situ* electrochemical deposition and demonstrated exceptional PEC activity in CO_2_ reduction and photodetection performance (Fig. 17c). Here, the as-prepared CuSe@BiOI exhibited a high current density of −57.9 mA cm^−2^ with over 80% selectivity for FA (Fig. 17d). According to the report, such enhancement in efficiency and selectivity was primarily attributed to the isotropic charge transport phenomenon where photogenerated charges were efficiently separated at the heterojunction interface due to the exposure of (102) crystal facets.

In another approach, crystal facet tuning is also considered to be responsible for influencing the formation of structural features such as crystal defects and O_Vac_.^271,272^ High-energy facets are often associated with unsaturated atomic coordination, which tends to generate defects such as step edges, dislocations and kinks, creating highly reactive sites. These defects act as catalytic hotspots by stabilizing key intermediates and promoting selective reaction pathways. Simultaneously, O_Vac_ are more prevalent on high-energy facets due to their inherent instability and tendency to lose lattice oxygen. These vacancies introduce localized electronic states within the bandgap, improving light absorption and facilitating charge carrier separation. In addition, O_Vac_ could also provide electron-rich centers that could improve adsorption and activation of oxygen-containing functional groups in biomass molecules. For example, a study demonstrates the role of facet tuning and O_Vac_ in optimizing electron transfer and ozone activation by synthesizing CeO_2_ with predominantly exposure of (110), (100), or (111) facets.^273^ In this study, it was found that CeO_2_ with dominant exposure of the (110) facet exhibited the highest efficiency for phenol mineralization due to its superior charge carrier separation and enhanced electron transfer for O_3_ activation. Interestingly, *ex situ*, quasi-situ, and *in situ* characterization revealed that facet modulation influences the electronic properties of CeO_2_ due to the increased number of O_Vac_ density, which is responsible for accelerating O_3_ activation and facilitating the formation of reactive oxygen species.

To summarize, crystal facet tuning plays a pivotal role in tailoring the catalytic performance of materials in PEC biomass and CO_2_ conversion by modulating the exposure of specific crystallographic planes. High-energy facets, characterized by unsaturated atomic sites, enhance reactant adsorption, lower activation barriers, and promote selective reaction pathways, while low-energy facets facilitate product desorption and improve selectivity. Moreover, crystal facet tuning significantly influences the electronic and optical properties of photocatalysts by optimizing charge carrier separation, modifying band structures, and amplifying localized electric fields, leading to enhanced PEC performance. Various studies have demonstrated how facet-dependent electronic structures, spatial charge separation, and defect formation contribute to improved reaction efficiencies. Additionally, the interplay between facet exposure and structural defects, such as O_Vac_, introduces reactive sites that further boost catalytic activity and selectivity. However, despite these advantages, several challenges limit the widespread application of facet tuning. Precisely controlling the synthesis of facet-exposed structures often requires complex, time-consuming, and condition-sensitive fabrication methods.^274,275^ These processes may have low scalability and reproducibility, hindering large-scale implementation. Furthermore, the interplay between multiple facets and their synergistic or antagonistic effects on complex biomass-derived intermediates remains difficult to predict and model, requiring advanced characterization and theoretical tools.^276^ All in all, while crystal facet tuning offers highly selective PEC systems, addressing its synthetic complexity and mechanistic uncertainties will be crucial for its practical deployment in biomass and CO_2_ valorization technologies. The utilization of crystal facet tuning for selective PEC biomass and CO_2_ valorization is summarized in Table 7.

**Table 7:** Summary of crystal facet tuning of photoelectrodes for PEC biomass valorization selectivity enhancement and tuning applications

Photoelectrode	Facet tuning function	Tuning method	Reaction	Target product(s)	Selectivity	FE	Reaction conditions	Ref.
(010)-Dominant BiVO_4_	Promotes glycerol adsorption, charge transport promoter, facilitating high carrier density	Solvent-assisted hydrothermal method	Glycerol oxidation	DHA	60%	N/A	• Electrolyte: Na_2_B_4_O_7_ (0.1 M) with glycerol (0.1 M)	259
							• Applied bias: 1.1 V_RHE_	
							• Light: AM 1.5G (100 mW cm^−2^)	
(002)-WO_3_	Lowering the energy barrier for oxidation, charge transport promoter	Solvothermal method	Glycerol oxidation	GA	73%	N/A	• Electrolyte: H_2_SO_4_ (0.5 M) with glycerol	260
							• Applied bias: 0.9 V_RHE_	
							• Light: AM 1.5G (100 mW cm^−2^)	
(102)-CuSe@BiOI	Charge separation promoter	Electrodeposition method	CO_2_ reduction	FA	80%	N/A	• Electrolyte: CO_2_-saturated KHCO_3_ (0.5 M)	270
							• Applied bias: 1.2 V_RHE_	
							• Light: AM 1.5G (150 mW cm^−2^)	

### 3.3. Defect engineering

Surficial vacancy and defect engineering have been rigorously studied to improve charge separation and surface reactivity for PEC reactions.^277–281^ Surface O_Vac_, for example, have been found to suppress surface charge recombination and improve charge transport of photoelectrodes.^282,283^ However, extra care is needed since bulk O_Vac_ have been shown to negatively impact the PEC performance due to the interruption of bulk charge transport.^47^ Other than O_Vac_, metal vacancies could be utilized to improve charge carrier mobility on the photoelectrode.^48^

**Fig. 18:**
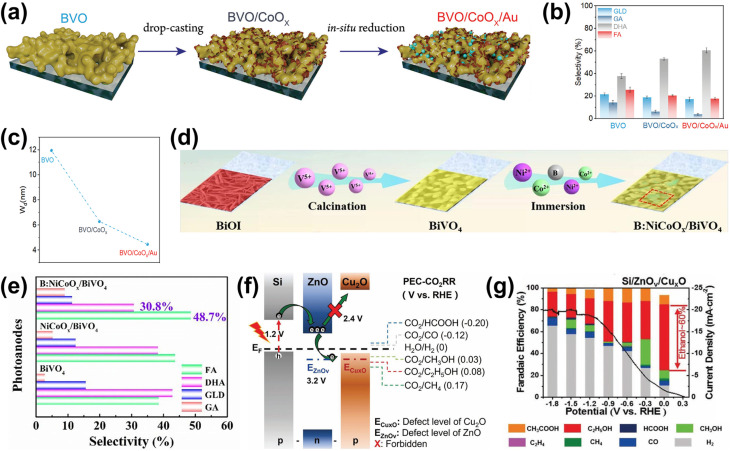
Improving charge transport through surficial defects for PEC biomass valorization selectivity enhancement. (a) Fabrication steps for the Au/CoO_*x*_/BiVO_4_ photoelectrode. A solution of Co(NO_3_)_2_·6H_2_O is mixed with ammonia solution and H_2_O_2_ and then stirred to oxidize Co^2+^ to Co^3+^. The mixture is subsequently drop-cast onto BiVO_4_ to form CoO_*x*_/BiVO_4_. (b) Increased selectivity towards DHA from PEC glycerol oxidation on Au/CoO_*x*_/BiVO_4_. (c) Depletion layer width comparison of Au/CoO_*x*_/BiVO_4_ compared to other photoelectrodes. Adapted from ref. 284 with permission. Copyright 2024 Wiley-VCH GmbH. (d) Preparation steps for the B:NiCoO_*x*_/BiVO_4_ photoelectrode. Pristine BiVO_4_ is immersed in a solution containing 50 mL NiCl_2_·6H_2_O (10 mM), 50 mL CoCl_2_·6H_2_O (10 mM), and NaBH_4_ for one hour. (e) Product selectivity of glycerol oxidation on the B:NiCoO_*x*_/BiVO_4_ photoelectrode. Adapted from ref. 285 with permission. Copyright 2024 Elsevier B.V. (f) Band alignment of the p-Si/ZnO_v_/Cu_*x*_O photoelectrode and electron flow schematics. (g) FE of the PEC CO_2_ reduction reaction on p-Si/ZnO_v_/Cu_*x*_O. Adapted from ref. 286 with permission. Copyright 2022 Wiley-VCH GmbH.

Introduction of vacancies and defects has also been used as a strategy to enhance PEC biomass valorization selectivity and reactivity through charge transport improvement. For instance, O_Vac_ on the CoO_*x*_/BiVO_4_ heterojunction of the Au/CoO_*x*_/BiVO_4_ photoelectrode can be utilized to enhance PEC glycerol valorization to high-valued DHA (Fig. 18a).^284^ This method enhances the glycerol-to-DHA selectivity from the initial 37.9% of pristine BiVO_4_ to 53.2% (Fig. 18b). The increased selectivity stems from the O_Vac_, which decreases the depletion layer width of the photoelectrode, from the initial 11.95 nm to 6.28 nm for CoO_*x*_/BiVO_4_ (Fig. 18c), indicating sharper band bending at the photoelectrode/electrolyte interface, which accelerates hole drift from the depletion region to the surface and suppresses charge recombination. Similarly, O_Vac_ are utilized in the ultrathin B-activated NiCoO_*x*_/BiVO_4_ photoelectrode (Fig. 18d).^285^ This treatment deposits a 2 nm thick B:NiCoO_*x*_ layer on BiVO_4_, providing abundant O_Vac_. The ultrathin-high O_Vac_ B:NiCoO_*x*_ layer creates active sites for H_2_O to form hydroxyl groups, which are then used to oxidize glycerol to FA. Furthermore, the O_Vac_ facilitates hole extraction or storage and accelerates highly oxidizing hole transport. With this approach, the system achieves a selectivity of 48.7% for 1.5 hours (Fig. 18e).

Additionally, the strategy of improving charge transport through defect engineering has also been pursued to obtain high-value products of PEC CO_2_ reduction with high efficiency. Considering the high value and energy density of multi-carbon products, the efficient production of ethanol and acetic acid is very attractive.^287^ However, to shift CO_2_ reduction selectivity towards multi-carbon products, the system needs to promote multi-electron transfer, which requires very efficient excitation and separation of multiple electrons. Thus, one example utilizes ZnO with O_Vac_ (ZnO_v_) and defective Cu_2_O (Cu_*x*_O) with p-Si to form a p–n–p band alignment (Fig. 18f).^286^ This alignment enables confining and accumulating multiple electrons in the conduction band of n-type ZnO_v_ under a built-in electric field, while its shallow energy level allows the electron to escape the well and into Cu_*x*_O. These tunneling defect energy levels on Cu_*x*_O are close to matching the energy levels of CO_2_-to-ethanol reduction. Therefore, the selectivity towards ethanol is improved (Fig. 18g). Notably, the architecture allows the system to reduce CO_2_ to ethanol at 0 V_RHE_.

**Fig. 19:**
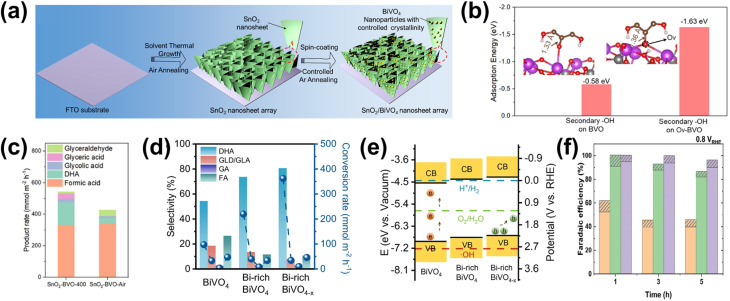
Surficial defects for selectivity tuning in PEC glycerol oxidation. (a) Synthesis steps for SnO_2_/O_Vac_-enriched BiVO_4_. (b) Calculated adsorption energy of secondary hydroxyl of glycerol on pristine and O_Vac_-enriched BiVO_4_. (c) Production rate of glycerol oxidation on SnO_2_/O_Vac_-enriched BiVO_4_ compared to SnO_2_/BiVO_4_ (SnO_2_/BiVO_4_-air). Adapted from ref. 288 with permission. Copyright 2025 The Royal Society of Chemistry. (d) PEC glycerol oxidation product selectivity on the Bi-rich BiVO_4−*x*_ photoelectrode compared to pristine BiVO_4_ and Bi-rich BiVO_4_. (e) Band alignments of the Bi-rich BiVO_4−*x*_ photoelectrode compared to other photoelectrodes. Adapted from ref. 289. Copyright 2024 The Author(s). (f) FE of glycerol oxidation products on WO_3_ with O_Vac_ (purple) compared with WO_3_ with fewer O_Vac_ (green) and pristine WO_3_ (orange). Adapted from ref. 290. Copyright 2024 The Author(s).

Aside from improving charge transfer to enhance the selectivity of PEC biomass valorization reactions, defects could serve as or form active sites for the adsorption of intermediates, which will contribute to the selectivity tuning of PEC biomass valorization.^47^ For instance, O_Vac_ on the surface of BiVO_4_ play a vital role in selectively adsorbing and activating secondary hydroxyl groups of glycerol molecules.^288^ BiVO_4_ was deposited through spin-coating on a SnO_2_ nanosheet array and further annealed in an Ar atmosphere to induce a higher concentration of O_Vac_ (Fig. 19a). The presence of O_Vac_ lowers the adsorption energy for secondary hydroxyls (Fig. 19b), thereby shifting the reaction selectivity towards DHA (Fig. 19c). However, the maximum DHA selectivity achieved is only up to 26.5%, with FA as one of the main products aside from DHA. The high FA production occurs due to the glycerol oxidation mechanism on the photoelectrode. The symmetrical molecules possess two primary hydroxyl groups, which when attacked by adsorbed *OH will oxidize to glyceraldehyde. Glyceraldehyde is prone to further oxidation to glyceric and glycolic acid, which through C–C cleavage can be converted into FA, thereby allowing substantial FA production.

Alternatively, a higher glycerol-to-DHA selectivity has been achieved by transforming a pristine monoclinic BiVO_4_ surface into a Bi-rich BiVO_4−*x*_ surface.^289^ Bi atoms have been shown to exhibit heightened adsorption capacity for the secondary hydroxyl group of glycerol, which is responsible for selective oxidation to DHA.^291^ Therefore, forming a Bi-rich surface layer will promote the glycerol oxidation pathway towards DHA. The method involves alkaline etching in NaOH to leach surface V atoms, followed by electrochemical etching in potassium borate (KBi) electrolyte to induce O_Vac_, providing more surface Bi atom active sites for glycerol adsorption. As shown in Fig. 19d, this approach improves selectivity towards DHA from 54% to 80.3%. Moreover, the improved selectivity also stems from the shifted band alignments toward the vacuum level (Fig. 19e), which is favorable for efficient electron–hole separation. Another example of leveraging surficial defects to tune PEC biomass valorization selectivity involves modulating O_Vac_ on the surface of a WO_3_ photoelectrode (WO_3−*x*_) through exposure to 15 mM NaBH_4_, generating exclusive surface defects.^290^ These vacancies increase the availability of W^5+^ active sites for glycerol adsorption. Using this method, the photoelectrode achieves 86.1% selectivity for glyceraldehyde with a production rate of 378.8 mmol m^−2^ h^−1^ (Fig. 19f). However, different from the previously mentioned interaction of the O_Vac_ with the glycerol molecules, the surface of WO_3−*x*_ has a lower adsorption and activation energy for the terminal hydroxyl group of the glycerol, which results in the favorable oxidation of glycerol to glyceraldehyde.

**Fig. 20:**
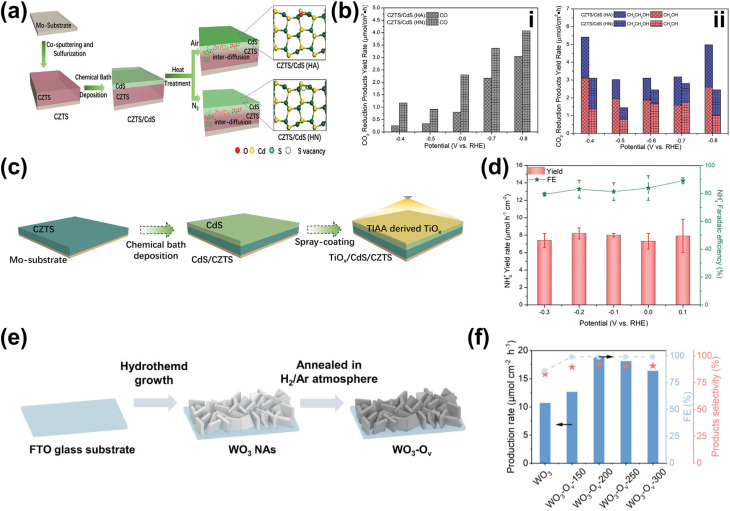
Surface defects in other PEC biomass valorization applications. (a) Preparation steps of the CZTS/CdS photoelectrode. (b) PEC CO_2_ reduction performance of CZTS/CdS photoelectrodes represented as yield for (i) CO and (ii) methanol and ethanol. Adapted from ref. 292 with permission. Copyright 2021 Wiley-VCH GmbH. (c) Fabrication steps of TiO_*x*_/CdS/CZTS. The TiO_*x*_ layer is deposited through spray-coating titanium diisopropoxide bis(acetylacetonate) (TIAA) on CdS/CZST. (d) PEC NO_*x*_ reduction performance of TiO_*x*_/CdS/CZTS represented as the yield and FE of ammonia as a function of applied bias. Adapted from ref. 293. Copyright 2022 The Author(s). (e) Fabrication process of O_Vac_-enriched WO_3_ (WO_3_-O_V_). (f) PEC procaine conversion to chloroprocaine performance of WO_3_-O_V_ photoelectrodes represented as production rate, FE, and selectivity at varying annealing temperatures. Adapted from ref. 294 with permission. Copyright 2024 American Institute of Chemical Engineers.

In another example, surface S vacancies on CdS/Cu_2_ZnSnS_4_ (CZTS) photoelectrodes can be modulated through heat treatment in varying ambient gases, demonstrating versatility in tuning PEC CO_2_ reduction selectivity (Fig. 20a).^292^ Heat treatment in air leads to a photoelectrode selective for methanol and ethanol, achieving yield rates of up to 3 μmol cm^−2^ h^−1^ and 2 μmol cm^−2^ h^−1^, respectively (Fig. 20b(i)). Conversely, heat treatment in N_2_ increased its selectivity for CO production up to 2.31 μmol cm^−2^ h^−1^ (Fig. 20b(ii)). This unique tunability arises from the S vacancies of the photoelectrode. The chemical bath deposition of the CdS layer on CZST induced S vacancies on the surface CdS layer. When treated in air, these vacancies are filled with O atoms, decreasing the S vacancies. This results in lower CO_2_ and CO adsorption energy. In contrast, when treated with N_2_, the CdS layer preserves the S vacancies. These vacancies facilitated CO desorption, therefore increasing its selectivity towards CO. Furthermore, O_Vac_ on the CdS/CZST photoelectrode can be modulated for NO_*x*_ reduction reaction selectivity tuning through the deposition of a temperature-sensitive TiO_*x*_ layer on CdS/CZST (Fig. 20c).^293^ In the deposition process, decomposition of acetylacetone is temperature sensitive and leads to the formation of Ti^3+^, which is closely related to the adsorption of NO_3_^−^ and *NO_2_^−^. The formation of Ti^3+^ on the surface accompanies the generation of O_Vac_ on TiO_*x*_. Therefore, the O defects can be modulated and indirectly tune the selectivity of the photoelectrode. When modified with TiO_*x*_ deposited at 250 °C, the photoelectrode achieved 89.1% FE towards ammonia (Fig. 20d). One of the underlying factors for the improved selectivity is the increase of catalytically active sites. The increase in surface coverage of Ti^3+^ proved to result in selectivity towards ammonia. Additionally, the AcAc ligand from TIAA is electrophilic, which promotes adsorption of NO_3_^−^ and NO_2_^−^, thereby facilitating ammonia production. In contrast, if the TiO_*x*_ layer on the photoelectrode is dominated by Ti^4+^ only, which is ordinary TiO_2_, it promotes NO_2_^−^ desorption before further oxidation, demonstrated by 60% faradaic efficiency at 0.0 V_RHE_ with no byproduct, resulting in 100% selectivity towards NO_2_^−^. It is worth highlighting that this method of surface engineering demonstrates a faradaic efficiency of 64.9% for ammonia upon testing with nitrate-rich wastewater, making it a potential candidate for nitrate-contaminated wastewater treatment.

Adding to the versatility of vacancy and defect utilization in PEC biomass valorization, O_Vac_-enriched WO_3_ can be utilized to improve the conversion of procaine to sustainably produce chloroprocaine, a valuable chemical for medical applications.^294^ The vacancies are induced through annealing in a H_2_/Ar atmosphere (Fig. 20e). Through this approach, the system is capable of converting procaine to chloroprocaine with a selectivity of up to 92% (Fig. 20f). This improved selectivity stems from the Cl^−^ ions that are preferably adsorbed onto the vacancy-enriched WO_3_, forming Cl^−^ enriched WO_3_ and facilitating the ions to participate in the production of chloroprocaine. Complementing the catalytic activity, the O_Vac_ also increases the light absorption and charge transport ability, which also contributes to increased selectivity. All in all, these examples demonstrate the crucial role that surficial vacancies and defects play in enhancing and tuning PEC biomass and CO_2_ valorization selectivity. By improving charge transfer and light absorption, as well as acting as and forming active sites for adsorption of reactants, surface vacancies and defects can selectively promote a specific reaction pathway, enhancing the selectivity. Despite these advantages, defect engineering also presents several limitations and challenges. Excessive or uncontrolled formation of defects, particularly in the bulk phase, can introduce trap states that hinder charge transport, reduce material stability, and cause undesirable side reactions.^295^ Moreover, the stability of defect-rich surfaces under prolonged PEC operation or in harsh reaction environments remains a concern, as vacancies may heal or evolve over time, leading to performance degradation.^296^ The synthesis of defect-engineered materials is also often sensitive to processing conditions, which complicates reproducibility and scalability.^297^ The utilization of surface vacancies and defects for PEC biomass and CO_2_ valorization selectivity control and enhancement is summarized in Table 8.

**Table 8:** Summary of surface defect utilization of photoelectrodes for PEC biomass valorization selectivity enhancement and tuning applications

Photoelectrode	Defect function	Induction method	Reaction	Target product(s)	Selectivity	FE	Reaction conditions	Ref.
Au/CoO_*x*_/BiVO_4_	Improving charge transfer by decreasing the depletion layer width	Ammonia and H_2_O_2_-assisted Co^2+^ oxidation, drop-casting	Glycerol oxidation	DHA	53.2%	N/A	• Electrolyte: H_2_SO_4_ (pH 2) with glycerol (0.5 M)	284
							• Applied bias: 1.2 V_RHE_	
							• Light: AM 1.5G (100 mW cm^−2^)	
B:NiCoO_*x*_/BiVO_4_	Facilitate hole extraction, storage, transfer, serving as active oxidation sites	Immersion in NaBH_4_-mixed solution	Glycerol oxidation	FA	48.7%	N/A	• Electrolyte: Na_2_SO_4_ (0.5 M) with glycerol (0.1 M)	285
							• Applied bias: 1.23 V_RHE_	
							• Light: AM 1.5G (100 mW cm^−2^)	
p-Si/n-ZnO_Vac_/p-Cu_*x*_O	Facilitating defect-assisted electron tunneling	Annealing in air and ion exchange in CuCl_2_	CO_2_ reduction	Ethanol	N/A	60%	• Electrolyte: CO_2_-dissolved KHCO_3_ (0.1 M)	286
							• Applied bias: −1.8 V_RHE_ to 0 V_RHE_	
							• Light: AM 1.5G (100 mW cm^−2^)	
SnO_2_/BVO-x	Secondary hydroxyl group adsorption sites	Annealing in an Ar atmosphere	Glycerol oxidation	DHA	26.5%	N/A	• Electrolyte: Na_2_SO_4_ (0.01 M) with glycerol (0.1 M)	288
							• Applied bias: 1.4 V_RHE_	
							• Light: 300 W Xe lamp (100 mW cm^−2^)	
Bi-rich BiVO_4−*x*_/BiVO_4_	Forming a Bi-rich surface with active sites for secondary hydroxyl adsorption	Chemical etching and electrochemical reduction	Glycerol oxidation	DHA	80.3%	N/A	• Electrolyte: Na_2_SO_4_ (0.01 M, pH adjusted to 2 with H_2_SO_4_) with glycerol (0.1 M)	289
							• Applied bias: 1.23 V_RHE_	
							• Light: AM 1.5G (100 mW cm^−2^)	
WO_3−*x*_	Adsorption and activation sites for terminal hydroxyl	Annealed in an Ar/O_2_ atmosphere and immersed in NaBH_4_	Glycerol oxidation	GLAD	86.1%	N/A	• Electrolyte: glycerol (2 M, pH 2)	290
							• Applied bias: 1.2 V_RHE_	
							• Light: AM 1.5G (100 mW cm^−2^)	
CZTS/CdS	Adsorption energy modulation	Chemical bath, heat treatment in air and N_2_ atmospheres	CO_2_ reduction	CO, ethanol and methanol	2.31 (CO), 3 (ethanol) and 2 μmol cm^−2^ h^−1^ (methanol) production yield	N/A	• Electrolyte: CO_2_-saturated KHCO_3_ (0.1 M)	292
							• Applied bias: −0.6 V_RHE_ (CO) and −0.4 V_RHE_ (ethanol and methanol)	
							• Light: AM 1.5G (100 mW cm^−2^)	
239 WO_3_-O_Vac_	Cl^−^ ion adsorption and Cl_2_ production sites	Annealed in an H_2_/Ar atmosphere	Procaine conversion	Chloroprocaine	N/A	92%	• Electrolyte: K_2_SO_4_ (0.5 M) or KCl	294
							• Applied bias: 1.0 V_RHE_	
							• Light: AM 1.5G (100 mW cm^−2^)	
Sn/TiO_2_/Si	CO_2_ molecule activation sites	Deposition of Sn	CO_2_ reduction	Formate	N/A	69%	• Electrolyte: CO_2_-saturated KHCO_3_	298
							• Applied bias: −1.0 V_RHE_	
							• Light: AM 1.5G (100 mW cm^−2^)	

### 3.4. Nanostructuring

**Fig. 21:**
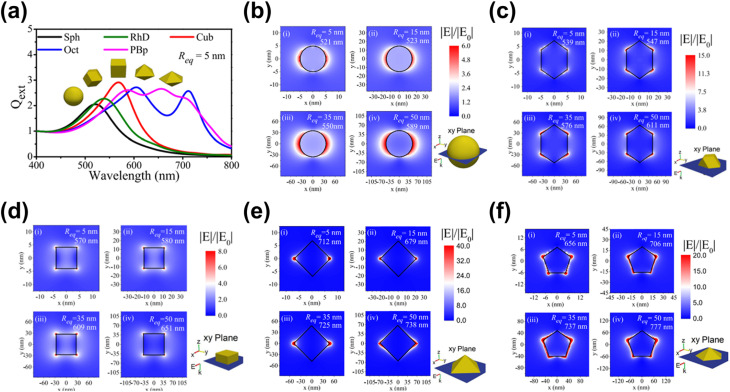
Nanostructuring effects on the optical properties of materials. (a) Extinction properties of Au nanoparticles with different shapes: sphere (Sph), rhombic dodecahedron (RhD), cube (Cub), octahedron (Oct), and pentagonal bipyramid (PBp). Distribution of near field electric enhancement in the *x*–*y* plane at different equivalent radii (*R*_eq_) of (b) Sph, (c) RhD, (d) Cub, (e) Oct, and (f) PBp Au nanoparticles. Optimizing nanoparticle shapes to achieve both the broadest light absorption and the highest, most widespread field enhancement is essential for enhancing the PEC performance of nanostructures. Adapted from ref. 310 with permission. Copyright 2019 American Chemical Society.

Aside from heterostructuring and surficial atomic modulation, nanostructuring has emerged as another common approach for selectivity control in PEC biomass valorization. Nanostructuring enables precise tuning of surface area,^299,300^ morphology,^301,302^ and active site distribution,^303,304^ which directly influences catalytic performance. Additionally, optimizing the shape and morphology of nanostructured materials enhances light harvesting, absorption, and scattering capabilities, further improving PEC performance.^305,306^ For example, in water splitting, the photocurrent density of TiO_2_ at 1.23 V_RHE_ varies significantly with structural complexity, increasing from 0D (0.02 mA cm^−2^), 1D (0.7 mA cm^−2^), 2D (0.44 mA cm^−2^), and 3D (2.59 mA cm^−2^).^307–309^ The enhanced performance of the 3D structure can be attributed to their ability to improve light absorption and scattering, particularly at sharp edges and corners, which serve as charge separation and accumulation sites. The sharp edges and corners in certain shapes, such as cubes, octahedrons, or pyramids, often serve as sites for charge separation and accumulation. Furthermore, nanostructuring plasmonic nanoparticles into varying shapes, such as spheres, rods, cubes, or dendritic structures, could provide unique localized surface plasmon resonance (LSPR) characteristics that can enhance light absorption and scattering. As a result, this enhancement would increase the local electromagnetic field near the catalyst surface, which could ultimately contribute to boosting photocatalytic activity and generating hotspots that can selectively drive specific reaction pathways. The influence of morphology on the near-electric field distribution of plasmonic nanoparticles has been demonstrated in various metal nanoparticles.^310–315^ Theoretical calculations show that the more localized the electrons or plasmons, the stronger the near-electric field, leading to brighter hotspots. As shown in Fig. 21a–f, among polyhedral Au nanoparticles, octahedral structures exhibit the highest electric field enhancement. However, the enhancement is concentrated in a relatively small region compared to other morphologies with a broader near-field distribution. Additionally, as nanoparticle shapes deviate from spherical forms, their absorption domain broadens, covering a wider wavelength range, which is advantageous for PEC biomass valorization.

**Fig. 22:**
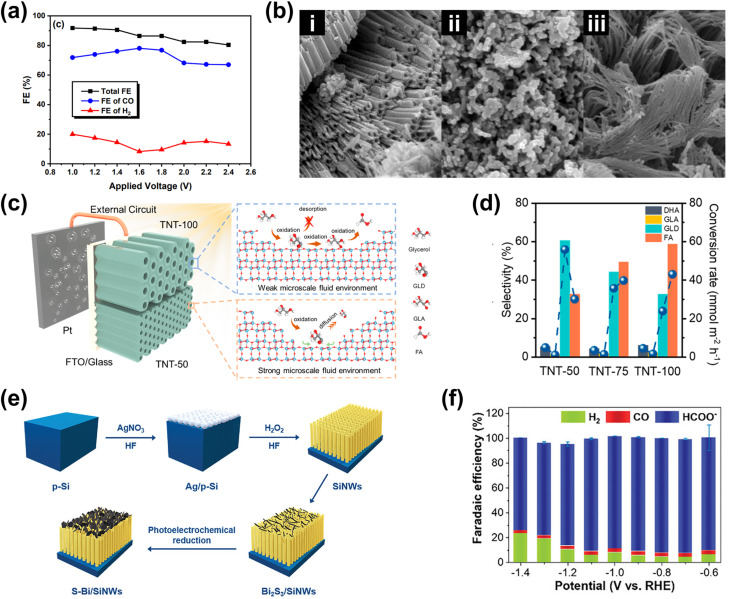
Nanostructuring for PEC biomass valorization selectivity tuning. (a) PEC CO_2_ reduction performance of nanostructured Ag nanocubes. Adapted from ref. 316 with permission. Copyright 2020 American Chemical Society. (b) Nanostructured TiO_2_ in the form of (i) nanotubes, (ii) nanosponges, and (iii) nanobelts for PEC glycerol oxidation. Adapted from ref. 321 with permission. Copyright 2022 Elsevier Ltd. (c) Architecture of inner diameter-modulated TiO_2_ nanotubes (TNTs) and illustration of the microscale fluid effect. (d) PEC glycerol oxidation product selectivity and conversion rate for inner diameter-modulated TNT photoelectrodes. Adapted from ref. 322 with permission. Copyright 2024 American Chemical Society. (e) Preparation steps for nanostructured S–Bi nanosheets on Si NWs. (f) PEC CO_2_ reduction product FE on the S–Bi nanosheet-deposited Si NW photoelectrode. Adapted from ref. 323. Copyright 2024 The Author(s).

Aside from leveraging the optical property tunability to improve PEC biomass valorization, nanostructuring also facilitates enhanced catalytic reactions by modifying surface interactions. For instance, nanostructuring Ag into Ag nanocubes with exposed (100) planes effectively suppresses H_2_ production by preferentially adsorbing CO_2_, thereby improving CO_2_ reduction selectivity towards CO (Fig. 22a).^316^ This demonstrates how tuning the nanoparticle shape and facet exposure can significantly impact selectivity in PEC processes. Moreover, the surface-to-volume ratio and active site density can be modulated by designing catalysts with unique nanoscale architectures such as nanorods, nanowires, or nanoporous structures.^317–320^ This approach creates microenvironments that enhance adsorption and activation of specific intermediates, steering reactions toward desired products. For example, TiO_2_ nanostructuring into nanotubes, nanosponges, and nanobelts (Fig. 22b(i–iii)) has been shown to improve conversion efficiency and selectivity in PEC glycerol oxidation, yielding value-added products such as glyceraldehyde (GLAD), dihydroxyacetone (DHA), and formic acid (FA).^321^

Furthermore, the geometric and spatial arrangement of nanoscale features could also impose steric constraints that favor certain reaction pathways while suppressing competing reactions. Consequently, this could also further contribute to the alteration of selectivity. Notably, for TiO_2_ nanotubes, it has been demonstrated that further nanostructuring, in the form of varying inner diameters, has a significant effect on the reaction kinetics, particularly for PEC glycerol oxidation.^322^ The microenvironment of nanotubes allows for the diffusion of molecules (Fig. 22c). In the case of decreasing the inner diameter of the nanotubes, the strength of the microenvironment, in the form of microscale fluid effect, is increased. Thus, the diffusion of the oxidation products is heightened, allowing the reaction to produce multi-carbon products. However, when the inner diameter is increased, this effect is weakened and the oxidation product desorption from the system is sluggish, allowing further oxidation. This unique nanostructuring-enabled reaction mechanism allows for selectivity tuning of the system to single-carbon products, FA, or multi-carbon products, GLAD (Fig. 22d).

We also note that nanostructuring could also facilitate efficient charge carrier separation and transport. This is primarily due to the reduction in the distance of photogenerated carriers and reaction sites, which are responsible for suppressing charge recombination.^236,324^ For instance, nanostructuring Bi_2_S_3_ to Bi nanoplates can help mitigate surface charge recombination of Si nanowires (NWs) (Fig. 22e).^323^ In multi-electron reactions, such as CO_2_ reduction, enlarging the junction area of semiconductors such as nanowires is expected to boost the photocurrent by reducing the travel distance of the minority carriers. However, excessive area augmentation can increase surface recombination, lowering open-circuit potential and reducing catalytic activity. In this approach, the S–Bi nanosheet rapidly extracts photogenerated electrons from the Si NWs and facilitates CO_2_ reduction on its surface. Therefore, with the enhanced charge transport, the photoelectrodes allow for improved selectivity towards formate (Fig. 22f).

In summary, nanostructuring is a powerful tool for improving and tuning PEC biomass and CO_2_ valorization selectivity through optical property modulation, electronic structure tailoring, and modifying interactions with reactants. By carefully nanostructuring photoelectrodes, researchers can manipulate optical, electronic, and catalytic properties to achieve higher efficiency and selectivity in PEC reactions. This approach not only enables precise control over reaction pathways but also paves the way for developing advanced photoelectrodes tailored for PEC biomass and CO_2_ valorization. Despite its significant advantages, nanostructuring also presents several challenges that must be carefully managed. Complex nanostructures often require intricate synthetic procedures, which can limit scalability, reproducibility, and cost-effectiveness.^325^ Stability is another key concern, as nanostructured materials with high surface energy or delicate morphologies may degrade or restructure under operational PEC conditions, diminishing long-term performance.^326^ Additionally, the interplay between morphology and selectivity is highly system-specific, and the mechanistic understanding of how nanostructure-induced microenvironments alter reaction kinetics remains incomplete.^327^ Therefore, achieving reproducible, durable, and mechanistically sound nanostructure designs requires careful balancing of structural complexity, material stability, and application-specific requirements. The utilization of nanostructuring for PEC biomass and CO_2_ valorization selectivity enhancement and tuning is summarized in Table 9.

**Table 9:** Summary of nanostructuring for PEC biomass valorization selectivity enhancement and tuning applications

Photoelectrode^a^	Role of nanostructuring	Method	Reaction	Target product(s)	Selectivity	FE	Reaction conditions	Ref.
Ag NCs/MCA	Increase CO_2_ adsorption by exposing the (100) plane	Sulfide-mediated polyol method	CO_2_ reduction	CO	N/A	80%	• Electrolyte: CO_2_-saturated KHCO_3_ (0.5 M)	316
							• Applied bias: 1.6 V	
							• Light: AM 1.5G (100 mW cm^−2^)	
NT-TiO_2_	Increasing active surface area	Anodic oxidation in a solution containing 2% volume of water and 98% volume of ethylene glycol and calcination	Glycerol oxidation	GLAD, DHA, and FA	31% (GLAD), 13% (DHA), and 5.7% (FA)	N/A	• Electrolyte: Na_2_SO_4_ (5 mM) with glycerol (10 mM)	321
							• Applied bias: 0.5 V_RHE_	
							• Light: 8 W UVA fluorescent lamp	
NS-TiO_2_	Increasing active surface area	Anodic oxidation in a solution containing 40% water and 60% glycerol and calcination	Glycerol oxidation	GLAD, DHA, and FA	32% (GLAD), 12% (DHA), and 6.7% (FA)	N/A	• Electrolyte: Na_2_SO_4_ (5 mM) with glycerol (10 mM)	321
							• Applied bias: 0.5 V_RHE_	
							• Light: 8 W UVA fluorescent lamp	
NB-TiO_2_	Increasing active surface area	Anodic oxidation in a solution containing 5% water and 95% glycerol and calcination	Glycerol oxidation	GLAD, DHA, and FA	31% (GLAD), 13% (DHA), and 7.0% (FA)	N/A	• Electrolyte: Na_2_SO_4_ (5 mM) with glycerol (10 mM)	321
							• Applied bias: 0.5 V_RHE_	
							• Light: 8 W UVA fluorescent lamp	
TNT-50	Modulating product diffusion through induced microscale fluid environments	Titanium foil anodization	Glycerol oxidation	GLAD	60.7%	N/A	• Electrolyte: Na_2_SO_4_ (0.5 M) with glycerol (0.1 M)	322
							• Applied bias: 1.23 V_RHE_	
							• Light: AM 1.5G (100 mW cm^−2^)	
TNT-100	Modulating product diffusion through induced microscale fluid environments	Titanium foil anodization	Glycerol oxidation	FA	60%	N/A	• Electrolyte: Na_2_SO_4_ (0.5 M) with glycerol (0.1 M)	322
							• Applied bias: 1.23 V_RHE_	
							• Light: AM 1.5G (100 mW cm^−2^)	
S–Bi/Si NWs	Improving charge transport from Si NWs	PEC reduction	CO_2_ reduction	Formate	N/A	93%	• Electrolyte: CO_2_-saturated KHCO_3_ (0.1 M)	323
							• Applied bias: −0.8 V_RHE_	
							• Light: AM 1.5G (100 mW cm^−2^)	
WO_3_/TiO_2_	Improves charge separation efficiency, extends light absorption, and provides adsorption and desorption sites	Hydrothermal	Glycerol oxidation	GLAD and DHA	61% (GLAD) and 92% (GLAD and DHA)	N/A	• Electrolyte: Na_2_SO_4_ (0.1 M) with glycerol (0.05 M)	328
							• Applied bias: 1.23 V_RHE_	
							• Light: AM 1.5G (100 mW cm^−2^)	
Au NW	Promoting adsorption of middle hydroxyl groups of glycerol	Electrodeposition	Glycerol oxidation	Lactic acid	80%	N/A	• Electrolyte: KOH (3 M) with glycerol	329
							• Applied bias: 0.95 V_RHE_	
							• Light: AM 1.5G (300 mW cm^−2^)	

a NC: nanocube, MCA: membrane cathode, NB: nanobelt, NS: nanosponge, NT: nanotube, NW: nanowire, and TNT: TiO_2_ nanotube.

## 4. Conclusions and outlook

The advancement of PEC biomass valorization has been significantly driven by surface engineering strategies, which play a crucial role in modulating the catalytic and electronic properties of photoelectrodes. Approaches such as surface functionalization, crystal facet tuning, defect engineering, and nanostructuring offer distinct advantages in enhancing selective PEC biomass valorization. In this review, we have comprehensively summarized and discussed how these strategies contribute to PEC biomass valorization selectivity through three key roles: first, they enhance charge generation, separation, and transport to reactants. Cocatalysts such as nanoparticles, quantum dots, and single-atom metals serve as electron or hole sinks, reducing recombination losses and directing carriers toward desired reaction sites. Second, they improve light absorption, particularly through surface functionalization, where cocatalysts, such as nanoparticles, quantum dots, and single-atom metals, serve as effective light harvesters. Surface decorations broaden the light absorption range through synergistic optical interactions. Plasmonic nanoparticles, quantum-confined dots, and tailored nanostructures increase photoresponse in the visible and near-infrared regions, while also amplifying local electromagnetic fields. Lastly, surface engineering plays a pivotal role in selectivity tuning by modulating photoelectrode-reactant interactions. Several studies have demonstrated that engineered surfaces can selectively generate active sites for specific molecules or key intermediates, thereby influencing reaction pathways. Among these strategies, crystal facet tuning and defect engineering, in particular, have demonstrated the ability to generate site-specific interactions, enabling high product selectivity.

Despite these advancements, several critical challenges must be addressed to fully realize the potential of surface engineering for scalable PEC biomass and CO_2_ valorization. One aspect is combining multiple surface engineering techniques, which could further open opportunities to amplify beneficial effects and achieve finer selectivity control.^330^ Furthermore, modified surfaces, in particular with molecular co-catalysts, often suffer from degradation under prolonged PEC operation. Additionally, nanostructured materials may degrade or restructure under operational PEC conditions. Addressing these stability issues will require the development of protective coatings, self-healing materials, or dynamically adaptive interfaces.^331–333^ Moreover, translating laboratory-scale strategies to large-scale practical systems demands scalable, cost-effective synthesis methods that maintain precise control over morphology, composition, and surface features.^334,335^ Simplifying complex fabrication processes without compromising performance is a key step toward commercial deployment, in particular for sophisticated surface engineering strategies such as single-atom metal. Additionally, deeper mechanistic insight into how surface modifications influence reaction intermediates, charge carrier dynamics, and microenvironmental conditions is essential. Advances in *operando* characterization and theoretical modeling will be instrumental in guiding rational design. Finally, to enable broader applicability, it is also essential to develop generalized surface engineering strategies that can be effectively applied to both oxidation and reduction half-reactions. Many current modifications are highly reaction-specific, and a more universal approach would facilitate the design of integrated PEC systems for complete biomass conversion or coupled CO_2_ reduction and biomass oxidation. All in all, by addressing current challenges, surface-engineered PEC systems can be further advanced into a more versatile and efficient platform for selective biomass and CO_2_ valorization.

## Author contributions

Y. T. A., F. F. A., and F. A. A. N. conceived the idea and defined the structure of the review. All authors contributed to the writing, reviewing, and editing of the manuscript. F. F. A. and F. A. A. N. supervised the overall project.

## Conflicts of interest

The authors declare no conflict of interest.

## Data Availability

No primary research results, software or code have been included in this review.
